# Chronic cholesterol depletion increases F-actin levels and induces cytoskeletal reorganization via a dual mechanism

**DOI:** 10.1016/j.jlr.2022.100206

**Published:** 2022-04-04

**Authors:** Parijat Sarkar, G. Aditya Kumar, Sandeep Shrivastava, Amitabha Chattopadhyay

**Affiliations:** CSIR-Centre for Cellular and Molecular Biology, Hyderabad, India

**Keywords:** cholesterol, statins, methyl-β-cyclodextrin, phosphatidylinositol, plasma membrane, F-actin, confocal microscopy, Rho GTPase, actin polymerization, MβCD, ABPs, actin binding proteins, CHO, Chinese hamster ovary, FDA, fluorescein diacetate, GPCR, G protein-coupled receptor, MβCD, methyl-β-cyclodextrin, MIP, maximum intensity projection, PI(4,5)P_2_, phosphatidylinositol 4,5-bisphosphate, PI, propidium iodide

## Abstract

Previous work from us and others has suggested that cholesterol is an important lipid in the context of the organization of the actin cytoskeleton. However, reorganization of the actin cytoskeleton upon modulation of membrane cholesterol is rarely addressed in the literature. In this work, we explored the signaling crosstalk between cholesterol and the actin cytoskeleton by using a high-resolution confocal microscopic approach to quantitatively measure changes in F-actin content upon cholesterol depletion. Our results show that F-actin content significantly increases upon chronic cholesterol depletion, but not during acute cholesterol depletion. In addition, utilizing inhibitors targeting the cholesterol biosynthetic pathway at different steps, we show that reorganization of the actin cytoskeleton could occur due to the synergistic effect of multiple pathways, including prenylated Rho GTPases and availability of membrane phosphatidylinositol 4,5-bisphosphate. These results constitute one of the first comprehensive dissections of the mechanistic basis underlying the interplay between cellular actin levels and cholesterol biosynthesis. We envision these results will be relevant for future understating of the remodeling of the actin cytoskeleton in pathological conditions with altered cholesterol.

## Graphical abstract

**Figure:**
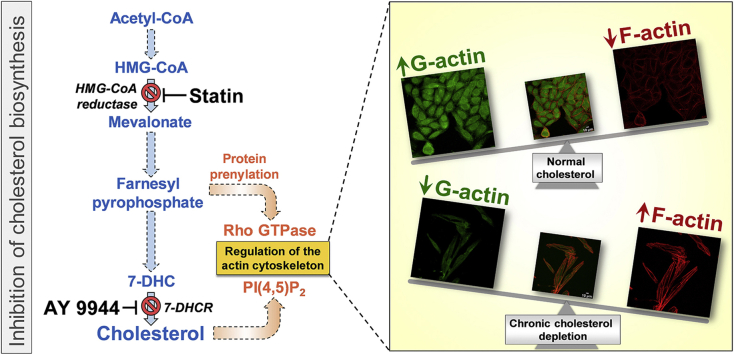

Plasma membranes are complex, quasi two-dimensional, noncovalent, organized assemblies of a diverse variety of lipids and proteins that allow confinement of intracellular contents and communication with the cellular exterior. They confer an identity to individual cells, besides providing an appropriate environment for proper functioning of membrane proteins. The understanding of biological membranes has evolved a long way from the fluid mosaic model proposed by Singer and Nicolson (1) to an immensely complex and dynamic macromolecular assembly of lipids and proteins (2). Cell membranes are often crowded (3), and the current understanding of their organization involves the concept of lateral heterogeneities, collectively termed as “membrane domains” (4, 5). These specialized regions are believed to be enriched in specific lipids (such as cholesterol and sphingolipids) and proteins supported by the underlying cytoskeleton and act as a platform for processes such as trafficking, sorting, signal transduction, and entry of pathogens over a wide range of spatiotemporal scales (6, 7, 8, 9).

The actin cytoskeletal network underlying the plasma membrane was initially not considered as a constituent of the plasma membrane (10). However, in the past few years, a number of observations using high-resolution microscopic techniques have established the concept of actin cytoskeleton dependent function and dynamics of membrane proteins (11, 12, 13, 14) and have led to the “anchored protein picket model” of membranes (15). In addition, a model involving crosstalk between membrane cholesterol and actin cytoskeleton is emerging based on observations such as reorganization of the cortical actin cytoskeleton due to depletion of plasma membrane cholesterol (16). Whereas evidence of direct interaction of membranes with the actin cytoskeleton is lacking, several cytoskeleton-associated proteins have been reported to act as adapters by simultaneously interacting with membrane lipids and the cytoskeleton (17, 18).

Actin is one of the most ubiquitous proteins in eukaryotic cells and exists in both monomeric (globular or G-actin) and polymeric (filamentous or F-actin) forms. The actin cytoskeleton is involved in a large number of cellular signaling events in addition to providing structural integrity to the cell (19). The two ends of F-actin are characterized by distinct growth rates; polymerization is relatively slow at the minus or pointed end and fast at the plus or barbed end (20). Cells maintain a dynamic actin cytoskeleton network by spatiotemporal regulation of the localization and function of a diverse collection of signaling, scaffolding, and actin binding proteins (ABPs) in response to a variety of stimulus (21). This provides a crucial mechanism so that dynamic changes in the actin cytoskeleton act as key regulators of cellular signaling (22, 23, 24, 25, 26). For this reason, malfunction of cytoskeletal proteins gets manifested as various diseases (27). Most ABPs are modular in nature which integrates actin binding, membrane binding, and signaling domains (28). In this context, Rho family GTPases have emerged as the largest group of proteins that mediate information transfer (signal transduction) from outside the cell to the cellular interior, which involves actin polymerization (elongation), severing, capping, and depolymerization (29).

Although the function of the actin cytoskeleton in cellular processes such as trafficking, motility, and endocytosis has been widely studied (30, 31), reorganization of the actin cytoskeleton upon modulation of membrane lipids is a relatively unexplored area. The interplay between membrane cholesterol and the underlying actin cytoskeleton is an emerging feature associated with the dynamic regulation of plasma membrane composition and the organization of constituent lipids (16, 32, 33). A major representative lipid in higher eukaryotic cellular membranes is cholesterol (34) which is biosynthesized as the end product of a long, multistep, and highly regulated enzymatic pathway (35, 36). Biosynthesis of cholesterol begins with acetyl-CoA that feeds into the mevalonate pathway to generate lanosterol. Cholesterol is synthesized from lanosterol either via desmosterol (the Bloch pathway) or via 7-dehydrocholesterol (the Kandutsch-Russel pathway) as immediate precursors (35, 37). It is noteworthy that one of the best-selling drugs in clinical history, statins, are cholesterol-lowering agents that act as competitive inhibitors of HMG-CoA reductase, the crucial enzyme catalyzing the rate-limiting step in the cholesterol biosynthesis pathway (38, 39, 40). Membrane lipids such as cholesterol and sphingolipids, along with the underlying actin cytoskeleton, assume significance owing to their ability to nonrandomly organize into domains in membranes that serve as hubs for cellular signaling originating from the plasma membrane (25, 41, 42). Importantly, cholesterol and the actin cytoskeleton have been shown to modulate the organization, dynamics, and function of membrane proteins such as G protein-coupled receptors (GPCRs) (43) which serve as major drug targets (44). In this context, a comprehensive understanding of the possible interplay between membrane cholesterol and the underlying actin cytoskeleton assumes significance.

In this overall context, we explored the effect of membrane cholesterol depletion on the regulation of cellular actin cytoskeleton dynamics by employing a quantitative confocal microscopy based approach. For this, we depleted membrane cholesterol from cells in a chronic fashion using statins and measured F-actin content, utilizing an approach previously developed in our laboratory (22), based on high-magnification imaging followed by image reconstruction. Our results suggest that F-actin content increases in response to chronic cholesterol depletion as a result of synergy between multiple pathways that are parallelly associated with cellular cholesterol biosynthesis. Notably, our results show that changes in F-actin are reversible in nature. Interestingly, acute cholesterol depletion had no significant effect on cellular F-actin, thereby highlighting the fine-tuned differences at a molecular level associated with different methods of cholesterol depletion. To the best of our knowledge, these results constitute the first comprehensive report quantifying dynamic changes in F-actin upon cholesterol depletion. We believe that dynamic reorganization of the actin cytoskeleton may represent an important determinant for membrane protein signaling in diseases such as Smith-Lemli-Opitz syndrome (SLOS) and desmosterolosis that are caused due to defects in cholesterol biosynthesis pathways (45, 46, 47).

## Materials and methods

### Materials

AY 9944, bovine serum albumin (BSA), CaCl_2_, cholesterol, cycloheximide, 1,2-dimyristoyl-*sn*-glycero-3-phosphocholine (DMPC), DMSO, EDTA, fluorescein diacetate (FDA), glycerol, gentamycin sulfate, methyl-β-cyclodextrin (MβCD), (±)-mevalonolactone, 3-(4,5-dimethylthiazol-2-yl)-2,5-diphenyl-tetrazolium bromide (MTT), MgCl_2_, MnCl_2,_ Na_2_HPO_4_, neomycin sulfate, penicillin, phenylmethylsulfonyl fluoride (PMSF), poly-l-lysine, propidium iodide (PI), sodium bicarbonate, staurosporine, streptomycin, Tris, Triton X-100, and Y27632 were obtained from Sigma Chemical Co. (St. Louis, MO). 1-Palmitoyl-2-oleoyl-*sn*-glycero-3-phosphocholine (POPC), 1-palmitoyl-2-oleoyl-*sn*-glycero-3-phosphoglycerol (POPG), 1-palmitoyl-2-oleoyl-*sn*-glycero-3-phosphoethanolamine (POPE), and porcine brain sphingomyelin lipids were obtained from Avanti Polar Lipids (Alabaster, AL). N*|*N,*O*-bis(trimethylsilyl)trifluoroacetamide was purchased from Supelco Analytical (Bellefonte, PA). DMEM/F-12 [Dulbecco’s modified Eagle’s medium/nutrient mixture F-12 (Ham) (1:1)], DMEM, and fetal calf serum (FCS) were from Gibco/Life Technologies (Grand Island, NY). ApoAlert Annexin V Apoptosis Kit was purchased from Clontech laboratories (Mountain View, CA). Bicinchoninic acid (BCA) reagent for protein estimation was from Pierce (Rockford, IL). Lovastatin, mevastatin, pravastatin, and simvastatin were obtained from Calbiochem (San Diego, CA). Alexa Fluor 488 conjugated DNase I, Alexa Fluor 546 conjugated phalloidin, Amplex Red Cholesterol Assay Kit, and jasplakinolide (Jas) were purchased from Molecular Probes/Invitrogen (Eugene, OR). Vectashield® Antifade Mounting Medium was obtained from Vector Laboratories (Burlingame, CA). Precoated silica gel 60 thin-layer chromatography (TLC) plates were from Merck (Darmstadt, Germany). All other chemicals used were of the highest available purity. Water was purified through a Millipore (Bedford, MA) Milli-Q system and used throughout.

### Cell culture and treatments

CHO-K1 cells were maintained in DMEM/F-12 (1:1) medium supplemented with 2.4 g/l sodium bicarbonate, 10% (v/v) FCS, 60 μg/ml penicillin, 50 μg/ml streptomycin, and 50 μg/ml gentamycin sulfate (complete DMEM/F-12 medium) in a humidified atmosphere with 5% CO_2_ at 37°C. HN2 and Neuro2a cells were grown in DMEM medium supplemented with 3.7 g/l sodium bicarbonate, 10% (v/v) FCS, 60 μg/ml penicillin, 50 μg/ml streptomycin, and 50 μg/ml gentamycin sulfate (complete DMEM medium) in a humidified atmosphere with 5% CO_2_ at 37°C. The stock solution of statins was prepared as described previously (48), added to cells grown for 24 h (final concentration 2.5–10 μM), and incubated in complete DMEM/F-12 (or complete DMEM in case of HN2 and Neuro2a cells) medium for 48 h in a humidified atmosphere with 5% CO_2_ at 37°C. Stock solution of AY 9944 was prepared in water, added to cells grown for 24 h (final concentration 2.5–10 μM), and incubated in DMEM/F-12 medium containing 5% (v/v) FCS for 60 h in a humidified atmosphere with 5% CO_2_ at 37°C. Control cells were grown under similar conditions without statin or AY 9944 treatments. Acute cholesterol depletion was carried out using MβCD as described previously (49). Briefly, cells were grown for 3 days followed by incubation in serum-free DMEM/F-12 medium for 3 h in a humidified atmosphere with 5% CO_2_ at 37°C. Cholesterol depletion was carried out by treating cells with increasing concentrations of MβCD in serum-free DMEM/F-12 medium for 30 min in a humidified atmosphere with 5% CO_2_ at 37°C, followed by washing with PBS. Cells treated with 10 mM MβCD were further grown in serum-free DMEM/F-12 medium for 2 h in a humidified atmosphere with 5% CO_2_ at 37°C. For mevalonate replenishment, a stock solution of 1 M mevalonate was prepared by hydrolyzing (±)-mevalonolactone with 1 M NaOH at 37°C for 1 h. Cells grown for 24 h in complete DMEM/F-12 medium were treated with 10 μM lovastatin in the presence of increasing concentrations of mevalonate for 48 h in a humidified atmosphere with 5% CO_2_ at 37°C. To sequester membrane phosphatidylinositol 4,5-bisphosphate (PI(4,5)P_2_), CHO-K1 cells were grown for 24 h in complete DMEM/F-12 medium supplemented with increasing concentrations of neomycin. Jas treatment was carried out for 30 min in a humidified atmosphere with 5% CO_2_ at 37°C. Stock solution of Jas was prepared in DMSO, and further concentration for treatment (0.1 μM) was prepared upon dilution of the stock in PBS containing 1 mM CaCl_2_ and 0.5 mM MgCl_2_ (buffer A). To block protein synthesis, the cell culture medium was supplemented with 1 μM cycloheximide during the last 24 h of lovastatin treatment. To explore the role of Rho-kinase (ROCK) in actin polymerization, cells were treated with 10 μM Y27632 in the presence of 10 μM lovastatin for 48 h or 10 mM MβCD for 30 min. In addition, cells treated with 10 mM MβCD were further cultured in serum-free DMEM/F-12 medium for 2 h with 10 μM Y27632.

### Metabolic replenishment of cholesterol with serum

After treatment with lovastatin for 48 h, cells were washed with PBS and grown in complete DMEM/F-12 for additional 24 h in a humidified atmosphere with 5% CO_2_ at 37°C.

### Apoptosis assay

The percentage of apoptotic cells was assessed by flow cytometry using an Annexin V-FITC/PI apoptosis detection kit as per the manufacturer's protocol. Briefly, after treatment with lovastatin for 48 h, cells were trypsinized (0.1% (w/v) trypsin-EDTA) and washed with complete DMEM/F-12 medium. Cells were further washed with 1x binding buffer and incubated with 5 μl of Annexin V-FITC (stock concentration 20 μg/ml) and 10 μl of PI (stock concentration 50 μg/ml) at a density of ∼5 × 10^6^ cells/ml in 1x binding buffer for 15 min at room temperature (∼23°C) in dark. Samples were analyzed using a Gallios flow cytometer (Beckman Coulter, Brea, CA). FITC and PI were excited at 488 nm, and emission was collected using 525/40 nm and 620/30 nm bandpass filters, respectively. As a positive control, CHO-K1 cells were treated with 5 μM staurosporine for 24 h in a humidified atmosphere with 5% CO_2_ at 37°C.

### Cell viability assay using live/dead staining

We assessed viability of cells utilizing a fluorescence-based dual-color labeling assay using FDA and PI, which exclusively stains viable cells and dead cells, respectively. FDA is taken up by live cells which convert the nonfluorescent FDA into fluorescent fluorescein. In contrast, PI cannot pass through a viable cell membrane and only enters into dead cells and subsequently labels nucleic acids. After treatment with lovastatin for 48 h (or AY 9944 for 60 h), cells were washed with PBS and labeled with 8 μg/ml of FDA and 20 μg/ml of PI in serum-free DMEM/F-12 medium for 10 min at room temperature (∼23°C) in dark. Cells were washed with PBS, and live cell imaging was carried out in serum-free DMEM/F-12 medium. Images were acquired on an inverted Zeiss LSM 880 confocal microscope (Jena, Germany) with an open pinhole. Fluorescein and PI were excited at 488 and 543 nm and emission was collected from 500 to 560 nm and 560 to 600 nm, respectively.

### MTT viability assay

Viability of cells in MβCD-treated conditions was determined using MTT assay (50). Briefly, ∼10^4^ cells were plated in a 96-well culture plate and acute cholesterol depletion was carried out as described above. After treatment, MTT (0.5 mg/ml in serum-free DMEM/F-12 medium) was added, and cells were further incubated for 3 h in a humidified atmosphere with 5% CO_2_ at 37°C. The growth medium was removed, and DMSO was used to dissolve the formazan crystals formed upon reduction of MTT salt by mitochondrial enzymes in live cells. Absorbance was measured at 540 nm using an EnSpire Multimode plate reader (PerkinElmer, Waltham, MA).

### Labeling of G- and F-actin

F-actin labeling was carried out as described previously (14, 22, 50). Briefly, cells were plated at a density of ∼10^4^ on poly-l-lysine-coated 22-mm glass coverslips and cholesterol depletion was carried out as described above. Cells were then washed with buffer A and fixed with ∼3.5% (w/v) formaldehyde in buffer A for 10 min at room temperature (∼23°C). Subsequently, permeabilization of cells was carried out in buffer A with Triton X-100 (0.5%. [v/v]) for 5 min at room temperature (∼23°C). Stock solutions of Alexa Fluor 546 conjugated phalloidin and Alexa Fluor 488 conjugated DNase I were prepared in methanol and PBS containing 50% (v/v) glycerol, respectively. After permeabilization, cells were washed and labeled with Alexa Fluor 546 conjugated phalloidin (final concentration: 0.3 μM) and Alexa Fluor 488 conjugated DNase I (final concentration: 0.17 μM) in buffer A for 1 h at room temperature (∼23°C) in dark. Since Jas has been previously shown to competitively inhibit binding of phalloidin to F-actin (22), cells (after permeabilization) were washed three times with buffer A upon Jas treatment before labeling with Alexa Fluor 546 conjugated phalloidin. After labeling, the coverslips were washed twice with PBS and mounted using Vectashield® antifade mounting medium. The edges of the coverslips were sealed with nail enamel and used for imaging. In case of neomycin treatment of cells, neomycin concentration was the same for washing, labeling, and mounting solutions.

### Confocal microscopy and F-actin quantitation

All images were acquired using an inverted Zeiss LSM 510 Meta or Zeiss LSM 880 confocal microscope (Jena, Germany). F-actin imaging was carried out by exciting Alexa Fluor 546 phalloidin at 561 nm and collecting emission from 575 to 630 nm. G-actin was imaged by exciting Alexa Fluor 488 DNase I at 488 nm and collecting emission from 500 to 550 nm. Quantitation of F-actin was carried out using a technique previously developed by us (22). Briefly, images of *z*-sections with a fixed step size of 0.32 μm were acquired with a 63×/1.4 NA oil immersion objective under 1 airy condition. Maximum intensity projections of 15 sections (∼4.8 μm from the base of the coverslip into the cellular interior) were generated, and a total cellular area of the projected images was determined manually using the software provided with a Zeiss LSM 510 Meta or Zeiss LSM 880 confocal microscope. Iso-surfaces (contours made upon joining voxels of equal fluorescence intensity across 15 *z*-sections) were generated from *z*-sections corresponding to each image using Imaris 8.4.0 software (Bitplane AG, Zurich, Switzerland). For obtaining iso-surfaces, we performed fluorescence intensity thresholding of *z*-sections followed by application of a Gaussian filter. The measured fluorescence volumes enclosed by iso-surfaces were normalized to the respected projected area of cells for a given field. G-actin was quantified using the same approach.

### Cell membrane preparation

Cell membranes were prepared as described previously (51). Briefly, after respective treatments, cells were harvested using ice-cold hypotonic buffer containing 10 mM Tris, 5 mM EDTA, and 0.1 mM PMSF, pH 7.4. Subsequently, cells were homogenized for 10 s at 4°C at maximum speed with a Polytron homogenizer. The obtained cell lysate was centrifuged at 500 *g* for 10 min at 4°C, and the resulting postnuclear supernatant was centrifuged at 40,000 *g* for 30 min at 4°C. The pellet containing membranes was suspended in 50 mM Tris buffer, pH 7.4, flash-frozen in liquid nitrogen, and stored at −80°C until further use. BCA assay was used to determine the total protein concentration in the isolated membranes (52).

### Estimation of cellular cholesterol content

Cell monolayers were washed twice with PBS after each treatment. Membrane cholesterol (unesterified) was estimated from cell lysates (in case of lovastatin-treated cells) or cell membranes (in case of MβCD-treated cells) using the Amplex Red cholesterol assay kit (53). Cholesterol values were normalized to total protein content estimated using BCA assay (52).

### Estimation of phospholipid content

Concentrations of phospholipids in cell membranes were estimated by digestion with perchloric acid (54) using Na_2_HPO_4_ as standard. We used 1,2-dimyristoyl-*sn*-glycero-3-phosphocholine as an internal standard to assess lipid digestion. Samples digested without perchloric acid showed negligible readings. Phospholipid values were normalized to the total protein content of cell membranes.

### Thin-layer chromatography

Lipid extraction from cell membranes obtained from control and cells treated with increasing concentrations of lovastatin was carried out according to the Bligh and Dyer method (55). Lipid extracts from ∼1 mg of protein were dried under a stream of argon at ∼40°C, and the dried lipids were dissolved in chloroform/methanol (1:1, v/v). Lipids were resolved on precoated silica gel TLC plates using chloroform/methanol/water (65:25:4, v/v/v) as the solvent system (56). Cholesterol, POPE, POPG, POPC, and sphingomyelin standards were run to identify the corresponding bands in lipid extracts obtained from control and lovastatin-treated cells. The separated lipids were visualized by charring with a solution containing 8% (v/v) phosphoric acid and 10% (w/v) cupric sulfate at ∼150°C (57).

### Gas chromatography and mass spectrometry

Cholesterol content in cell lysates treated with AY 9944 was quantitated using gas chromatography-mass spectrometry (GC-MS). GC-MS analysis was carried out using an Agilent 6890N gas chromatograph (Agilent Technologies, Palo Alto, CA) equipped with a model 5973i mass selective detector and 7683 series injector. For sterol measurements, cellular lysates were prepared and lipids were extracted according to the Bligh and Dyer method (55), after the addition of 1 mg of coprostanol as an internal recovery standard. The extracted chloroform phase containing lipids was dried under nitrogen. The lipid extract was hydrolyzed in 1 M NaOH in ethanol for 1 h at 70°C, extracted with *n*-hexane, and subsequently converted into trimethylsilyl ether derivatives followed by injection into a capillary column. Sterols were identified and quantitated using GC-MS as described previously (58). The absolute concentrations of various sterols in the samples were calculated from the calibration curves obtained using pure standards. The concentration of coprostanol was used to calculate the recovery of the sterols.

### Statistical analysis

GraphPad Prism software version 4.0 (San Diego, CA) was used to estimate significance levels using Student’s two-tailed unpaired *t-*test. Plots were generated using Microcal Origin software, version 9.7 (OriginLab, Northampton, MA).

## Results

### Manipulation of membrane cholesterol using chronic depletion

In order to explore the role of membrane cholesterol in the dynamics of the actin cytoskeleton, we depleted cholesterol from CHO-K1 cells in a chronic fashion. Unlike acute depletion methods (such as extraction using MβCD), chronic cholesterol depletion occurs over a longer time scale that mimics physiological conditions (59). In this context, statins have emerged as best-selling oral cholesterol-lowering drugs that have been extensively used to lower cellular cholesterol levels (39, 40). Statins are competitive inhibitors of HMG-CoA reductase, the rate-limiting enzyme in the cellular cholesterol biosynthesis pathway (38).

**Fig. 1:**
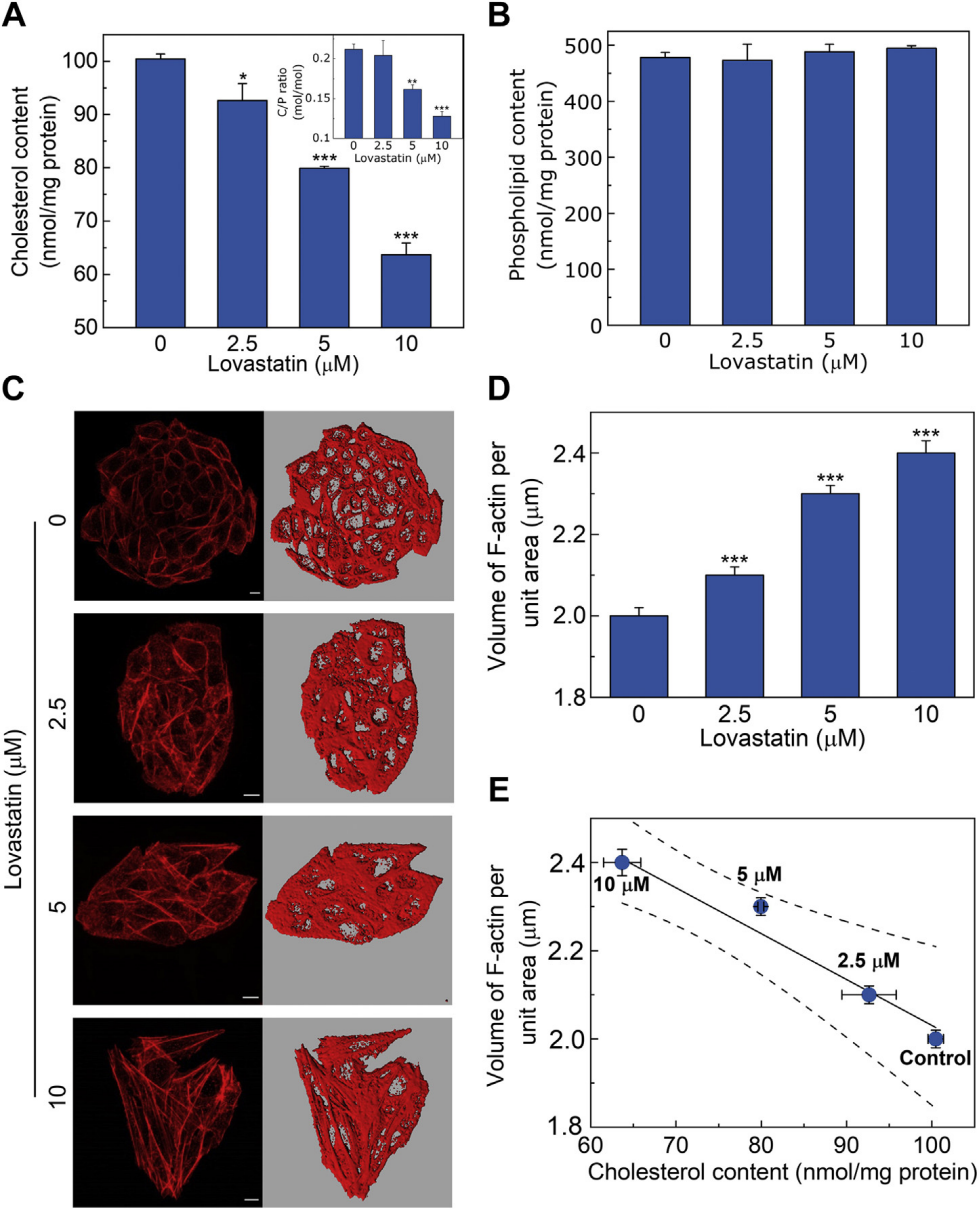
Chronic cholesterol depletion leads to polymerization of actin cytoskeleton. A: Membrane cholesterol content of CHO-K1 cells treated with increasing concentrations of lovastatin. Values are expressed as absolute cholesterol content in cell membranes and are normalized to protein content. Data represent means ± SEM of three independent experiments (∗ and ∗∗∗ correspond to a significant difference [*P* < 0.05 and *P* < 0.001] in membrane cholesterol content in lovastatin-treated cells relative to untreated cells). The inset shows cholesterol/phospholipid ratio (C/P, mol/mol) under these conditions. Data represent means ± SEM of three independent experiments (∗∗ and ∗∗∗ correspond to a significant difference [*P* < 0.01 and *P* < 0.001] in C/P ratio in lovastatin-treated cells relative to untreated cells). B: Membrane phospholipid content of CHO-K1 cells treated with increasing concentrations of lovastatin. The invariance in phospholipid content in cholesterol-depleted cells indicates that treatment with lovastatin is specific to cholesterol. Values are expressed as absolute phospholipid content in cell membranes and are normalized to protein content. Data represent means ± SEM of four independent experiments. C: Representative confocal micrographs showing organization of F-actin in control and cells treated with increasing concentrations of lovastatin. F-actin was labeled with Alexa Fluor 546 conjugated phalloidin. The MIPs of 15 *z*-sections from the base of the coverslip (∼4.8 μm from the base into the cell) are shown in left panels. Increase in F-actin filaments can be observed upon treatment with increasing concentrations of lovastatin. Panels on the right represent the iso-surfaces (defined as voxel contours of equal fluorescence intensity) generated from the *z*-sections corresponding to MIPs shown in the respective left panels. The scale bars represent 10 μm. Values obtained upon quantitation of F-actin in control and cholesterol-depleted cells are shown in panel (D). To quantify the F-actin content, the cellular volume enclosed by the iso-surface was normalized to the projected area of cells. Data represent means ± SEM of ∼40 different fields from at least five independent experiments (∗∗∗ corresponds to a significant [*P* < 0.001] difference in F-actin content in cholesterol-depleted cells relative to untreated cells). E: Correlation between membrane cholesterol content and F-actin levels in CHO-K1 cells upon chronic cholesterol depletion with increasing concentrations of lovastatin. Values of cholesterol content and corresponding values of F-actin content are taken from Fig. 1A, D, respectively. Linear regression analysis (plotted as a solid line) yielded a correlation coefficient (r) ∼0.97. The significance of the correlation between membrane cholesterol content and F-actin level is apparent from the 95% confidence band (plotted as dashed lines). See Materials and methods for more details.

Figure 1A shows membrane cholesterol content in cells treated with increasing concentrations of lovastatin for 48 h. The figure shows a dose-dependent reduction in membrane cholesterol content, with maximum reduction (∼36%) upon treatment with 10 μM lovastatin. Importantly, changes in membrane phospholipid content under similar conditions were negligible (Fig. 1B), indicating that lovastatin treatment is specific to cholesterol. In addition, membrane lipid contents of control and lovastatin-treated cells were analyzed by TLC and showed no changes in phospholipid composition upon chronic cholesterol depletion (see supplemental Fig. S1). The cholesterol/phospholipid ratio (C/P ratio) in the plasma membrane is an important marker for several pathophysiological conditions (60). C/P ratio upon chronic cholesterol depletion is shown as an inset in Fig. 1A and displays a similar trend as total membrane cholesterol content (Fig. 1A). C/P ratio was found to be ∼0.21 (mol/mol) in untreated cells, and this ratio is reduced to ∼0.13 (mol/mol) when cells were treated with 10 μM lovastatin. Importantly, control experiments showed that cells were viable even at the highest concentration of lovastatin (supplemental Fig. S2). We found that treatment with higher concentrations of lovastatin (e.g., 15 μM) in CHO-K1 cells led to compromised cell viability (apparent from the appearance of PI labeling, see supplemental Fig. S2). We therefore chose an optimal concentration range (2.5–10 μM) of lovastatin to retain cellular viability during subsequent experiments. Interestingly, lovastatin is known to act as an inducer of apoptosis in mammalian cell lines (61). We therefore ensured that our experimental conditions did not trigger apoptosis (assessed by phosphatidylserine externalization) in CHO-K1 cells (supplemental Fig. S3).

### Chronic cholesterol depletion leads to actin polymerization in CHO-K1 cells

To explore possible reorganization of actin cytoskeleton as a consequence of chronic cholesterol depletion, it is crucial to quantitatively measure the amount of F-actin under these conditions. Unfortunately, intensity-based analysis for quantitating F-actin is often complicated (62) by the fact that F-actin fragments and aggregates appear brighter under a fluorescence microscope (14). To circumvent this issue, we previously developed a quantitative high-resolution confocal microscopy based approach that allows measurements of F-actin content using an image reconstruction method (22). To quantitatively estimate the extent of F-actin reorganization, we treated cells with increasing concentrations of lovastatin and labeled F-actin with Alexa Fluor 546 conjugated phalloidin (a specific fluorescent probe for F-actin, (63)). Figure 1C shows representative confocal micrographs with maximum intensity projections (MIPs) of the actin cytoskeleton upon chronic cholesterol depletion. As qualitatively evident from the figure, treatment of CHO-K1 cells with lovastatin resulted in polymerization of F-actin, and consequently an increase in F-actin was observed under these conditions (compare untreated vs. 10 μM lovastatin-treated conditions in the left panel of Fig. 1C). The right panels in Fig. 1C represent iso-surface maps (defined as contours generated upon joining voxels of equal fluorescence intensity) of cellular F-actin from confocal *z*-sections corresponding to the projected images shown in the respective left panels. In order to quantitatively estimate cellular F-actin subsequent to chronic cholesterol depletion, we normalized the volume enclosed by the iso-surface in each case to the projected area of cells. This ratio (fluorescence volume/area of cell(s)) serves as a faithful indicator of the F-actin content in cells. The F-actin content quantitated this way is shown in Fig. 1D. With increasing cholesterol depletion using lovastatin, we observed a progressive increase in cellular F-actin content. The highest concentration of lovastatin (10 μM) resulted in ∼20% increase in F-actin content (Fig. 1D). Interestingly, chronic cholesterol depletion was accompanied by a drastic change in overall cellular morphology (supplemental Fig. S4). Cholesterol-depleted cells adopted an elongated shape at high concentrations of lovastatin, which could be due to formation of longer F-actin filaments under these conditions. To examine a possible correlation between the increase in cellular F-actin content and corresponding decrease in membrane cholesterol level, we plotted F-actin content (from Fig. 1D) versus membrane cholesterol level (from Fig. 1A). The resultant plot is shown in Fig. 1E. We performed a linear regression analysis between F-actin content and membrane cholesterol level and obtained a negative correlation of ∼0.97. Importantly, the 95% confidence intervals contained all data points, thereby implying a close relationship between the two parameters observed. Such a tight correlation between membrane cholesterol level and F-actin content implies a distinct molecular basis of regulation of actin cytoskeleton by membrane cholesterol.

In a control experiment, we explored the mutual dependence between F-actin levels and membrane cholesterol content by first inducing actin reorganization using Jas and then measuring membrane cholesterol level at different time intervals after Jas treatment (supplemental Fig. S5). Jas is known to induce actin polymerization and stabilize actin filaments (64). Interestingly, actin polymerization (see supplemental Fig. S5A) did not affect cholesterol content of cell membranes (supplemental Fig. S5B), thereby suggesting that membrane cholesterol level is not sensitive to polymerization of the actin cytoskeleton.

### Actin polymerization is due to regulation within the cholesterol biosynthetic pathway

**Fig. 2:**
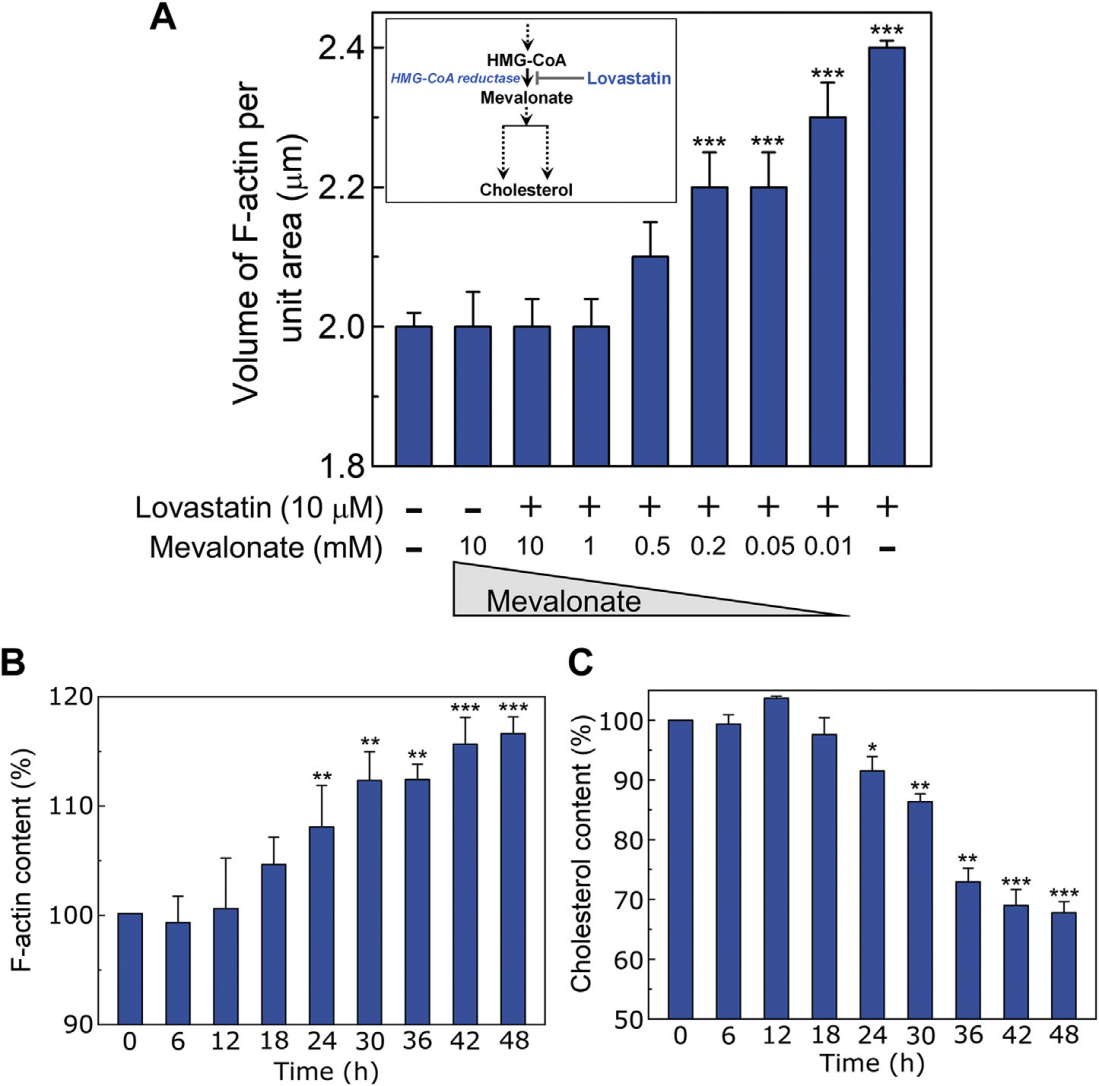
Altered actin cytoskeleton dynamics upon chronic cholesterol depletion is due to regulation within the cholesterol biosynthetic pathway. A: CHO-K1 cells were treated with 10 μM lovastatin alone or in combination with increasing concentrations of mevalonate (10 μM - 10 mM) and subsequently F-actin was quantified as described in Fig. 1. Restoring the mevalonate pathway by treatment of lovastatin in the presence of mevalonate inhibited actin polymerization in a dose-dependent manner. The inset shows key steps in the cholesterol biosynthesis pathway and the site of action of lovastatin. Data represent means ± SEM of ∼10 different fields from three independent experiments (∗∗∗ corresponds to a significant ([*P* < 0.001] difference in F-actin content in cells treated with lovastatin alone [or lovastatin in the presence of mevalonate] relative to untreated cells). B: Time course of actin polymerization in response to chronic cholesterol depletion. CHO-K1 cells were treated with 10 μM lovastatin with increasing incubation time, fixed at specified time intervals, and F-actin was labeled and quantified as described in Fig. 1. Values shown are normalized to F-actin levels in untreated (0 h) cells. Data represent means ± SEM of ∼10 different fields from three independent experiments (∗∗ and ∗∗∗ correspond to significant [*P* < 0.01 and *P* < 0.001, respectively] differences in F-actin content associated with cholesterol-depleted cells relative to control cells). C: Membrane cholesterol content of CHO-K1 cells treated with 10 μM lovastatin with increasing incubation time. Values shown are normalized to cholesterol content in untreated (0 h) cells. Data represent means ± SEM of three independent experiments (∗, ∗∗, and ∗∗∗ correspond to significant [*P* < 0.05, *P* < 0.01, and *P* < 0.001, respectively] differences in cholesterol content associated with cholesterol-depleted cells relative to control cells). See Materials and methods for more details.

Chronic cholesterol depletion using statin is often complicated due to pleiotropic effects independent of cholesterol lowering by inhibition of HMG-CoA reductase (65, 66). We therefore explored whether lovastatin-mediated actin polymerization could be reversed by supplementation of intermediates from the cholesterol biosynthetic pathway. For this, we treated CHO-K1 cells with 10 μM lovastatin in the presence of increasing concentrations of mevalonate, the precursor of cholesterol and other isoprenoids. Figure 2A shows that cotreatment of cells with lovastatin and mevalonate reversed actin polymerization in a dose-dependent fashion, suggesting that mevalonate could over-ride the inhibitory effects of lovastatin. These results confirm that actin polymerization upon chronic cholesterol depletion is due to regulation within the cholesterol biosynthetic pathway.

### Kinetics of actin polymerization

In order to monitor the kinetics of the polymerization process under chronic cholesterol depletion, we treated CHO-K1 cells with 10 μM lovastatin with increasing time of exposure and measured F-actin levels (Figs. 2B and S6). Figure 2B shows that there was no significant change in F-actin levels during initial 18 h of lovastatin treatment. The increase in F-actin levels post 18 h of treatment marks the beginning of actin polymerization upon chronic cholesterol depletion. The plateauing nature of the increase beyond 42 h represents an upper limit of the F-actin content beyond which we did not observe any increase in F-actin levels (Fig. 2B). Interestingly, we observed a progressive decrease in membrane cholesterol content in the presence of 10 μM lovastatin (Fig. 2C), further highlighting the inverse correlation between membrane cholesterol content and the level of cellular F-actin.

### Actin polymerization is not specific to a particular statin or cell type

Statins are available in various forms, all of which share a common HMG-like moiety (therefore competitively inhibit the entry of HMG-CoA to the active site of the enzyme HMG-CoA reductase) and rigid, hydrophobic groups that are covalently attached to the HMG-like moiety (38). Statins are characterized by their unique lipophilicity (based on octanol/water partition coefficient [P_O/W_]) that determines its ability to cross the blood-brain-barrier (67, 68, 69). To check the specificity of statins in inducing actin polymerization, we additionally utilized three different statins (pravastatin, mevastatin, and simvastatin) for our work. These statins have varying lipophilicity (P_O/W_ in the order: pravastatin << mevastatin < lovastatin < simvastatin), although with similar inhibition constants in nanomolar range (38). We observed that all three statins (in addition to lovastatin, as described above) exhibit an actin polymerizing effect in CHO-K1 cells (supplemental Fig. S7). In addition, supplemental Fig. S7B, D, F shows that the extent of increase in F-actin content was comparable across different statins, thereby suggesting that cholesterol lowering (and not the chemical nature of statin) is fundamental to dynamic changes in the cytoskeleton. Interestingly, human brain is highly enriched in cholesterol and the largest cholesterol pool of the body exists in the brain (70, 71). Although the central nervous system accounts for ∼2% of the body mass, it contains ∼25% of cholesterol of the body (70, 71). Cholesterol does not cross the blood-brain-barrier and is synthesized in the brain in situ with minimum losses to circulation (70, 72). Statins are known to cross the blood-brain-barrier based on their lipophilicity, and therefore, the lipophilic statins (e.g., simvastatin and lovastatin) are most effective in reducing brain cholesterol (69). Keeping this in mind, we tested the effect of cholesterol depletion on F-actin levels in neuronal cells. For this, we utilized HN2 (a hybrid cell line between hippocampal cells and mouse neuroblastoma (73)) and Neuro2a (mouse neuroblastoma cells (74)) cells for our actin quantification measurements (supplemental Fig. S8). Our results showed that the increase in F-actin content in neuronal cells displayed a trend similar to CHO-K1 cells (supplemental Fig. S8B, C). These results suggest that the observed increase in F-actin levels upon chronic cholesterol depletion is not a specific feature of CHO-K1 cells.

### Chronic cholesterol depletion affects G-actin/F-actin ratio

**Fig. 3:**
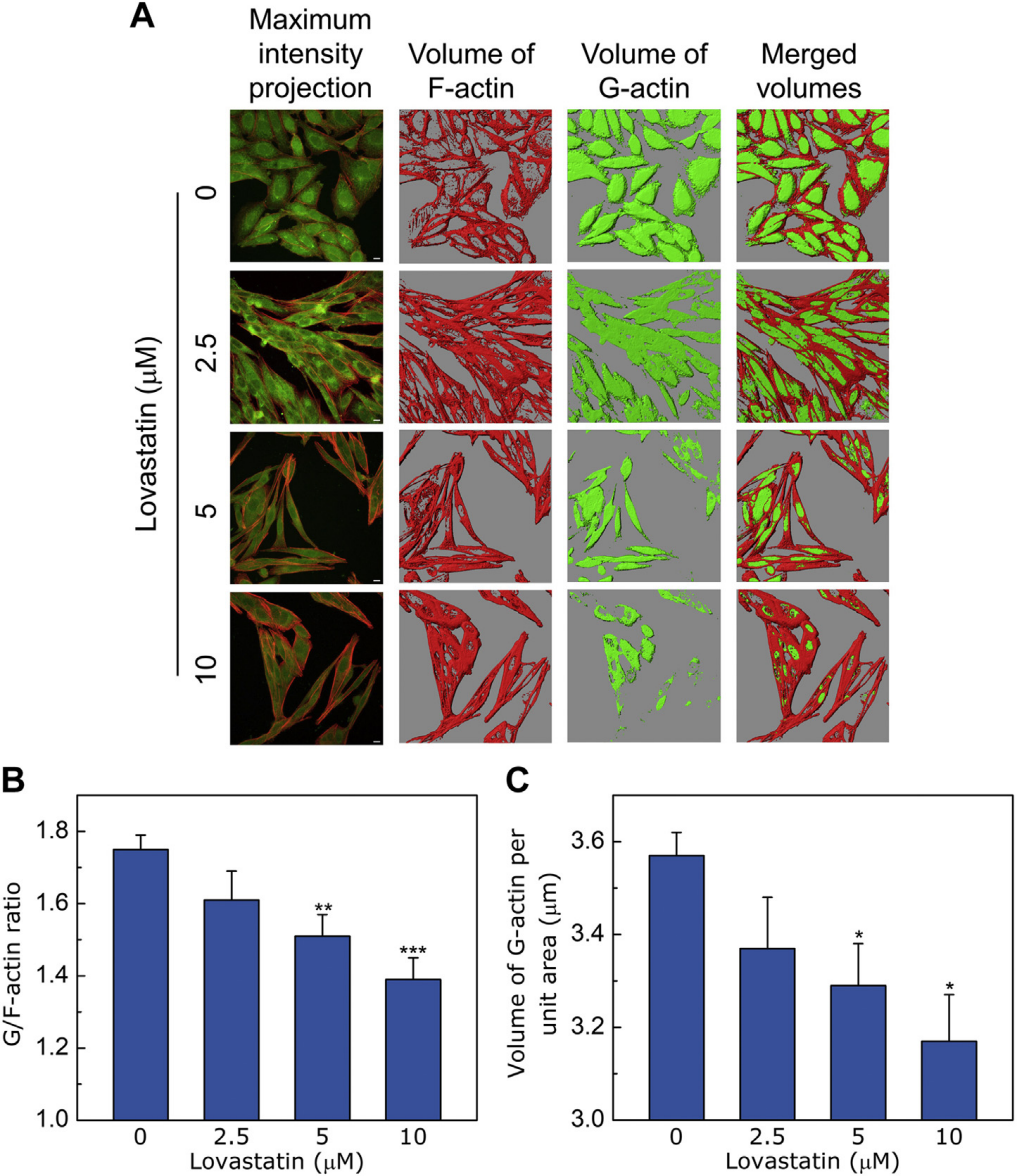
Increases in F-actin levels are associated with a concomitant decrease in G-actin content. A: Overall organization of the actin cytoskeleton in CHO-K1 cells treated with increasing concentrations of lovastatin. F- and G-actin were labeled with Alexa Fluor 546 conjugated phalloidin (red) and Alexa Fluor 488 conjugated DNase I (green), respectively. The scale bars represent 10 μm. Chronic cholesterol depletion using increasing concentrations of lovastatin leads to progressive reduction in G-actin iso-surface and simultaneous increase in F-actin iso-surface. The G-/F-actin ratio and G-actin content obtained upon quantitation of G- and F-actin in control and cholesterol-depleted cells are shown in panels B and C, respectively. The cellular volumes enclosed by the corresponding iso-surfaces were normalized to the projected area of cells calculated (as described in Fig. 1). Data represent means ± SEM of ∼10 different fields from at least three independent experiments (∗, ∗∗, and ∗∗∗ correspond to a significant [*P* < 0.05, *P* < 0.01, and *P* < 0.001, respectively] difference in G-/F-actin ratio [or G-actin content] in cholesterol-depleted cells relative to control cells). See Materials and methods for more details.

Actin is one of the most abundant proteins in eukaryotic cells and exists in both monomer (globular or G-actin) and polymer (filamentous or F-actin) forms (21, 75). In cells, soluble G-actin is maintained in a dynamic equilibrium with F-actin and the extent of actin polymerization and depolymerization is orchestrated by a number of ABPs in response to various stimuli (21). Such a tunable ratio of G-/F-actin offers a key cellular trigger that leads to dynamic changes in the cytoskeletal network which acts as a transducer of signaling transients (14, 23, 24, 26). In order to monitor the effect of chronic cholesterol depletion on the G-/F-actin ratio in a quantitative fashion, we simultaneously labeled F- and G-actin utilizing Alexa Fluor 546 conjugated phalloidin and Alexa Fluor 488 conjugated DNase I (a specific probe of G-actin (76)) (Fig. 3A). The first panel on extreme left (denoted as MIP) in Fig. 3A shows representative MIPs from control and cholesterol-depleted cells illustrating the overall organization of the actin cytoskeleton. The volume enclosed by the iso-surface corresponding to F-actin (shown in red) displayed a progressive increase, whereas the volume enclosed by the iso-surface corresponding to G-actin (shown in green) showed a progressive reduction upon treatment with increasing concentrations of lovastatin. To quantitatively estimate G-/F-actin ratio under these conditions, we plotted the ratio of the volumes enclosed by the iso-surfaces in each case (shown in Fig. 3B). The figure shows that increasing cholesterol depletion resulted in a progressive reduction of G-/F-actin ratio (Fig. 3B) with a concomitant reduction in G-actin content (see Fig. 3C). These results highlight a possible perturbation of the equilibrium between G- and F-actin in cholesterol-depleted cells, where F-actin filaments are formed using the available cytosolic pool of monomeric G-actin. Interestingly, we observed a larger variation in G-actin content across cells (as apparent from large error bars in Fig. 3C), possibly indicating intrinsic heterogeneity in G-actin levels in different cells. To check whether newly synthesized proteins are required for the polymerization of F-actin, we carried out chronic cholesterol depletion in the presence of protein translation blocker, cycloheximide (77), and quantified F-actin levels (supplemental Fig. S9). We did not observe any significant difference in F-actin levels in the presence of cycloheximide for both control and lovastatin-treated cells (supplemental Fig. S9B, C), which further reinforces that the existing pool of G-actin is utilized for the polymerization process during lovastatin treatment.

### Chronic cholesterol depletion induced actin polymerization is reversible in nature

**Fig. 4:**
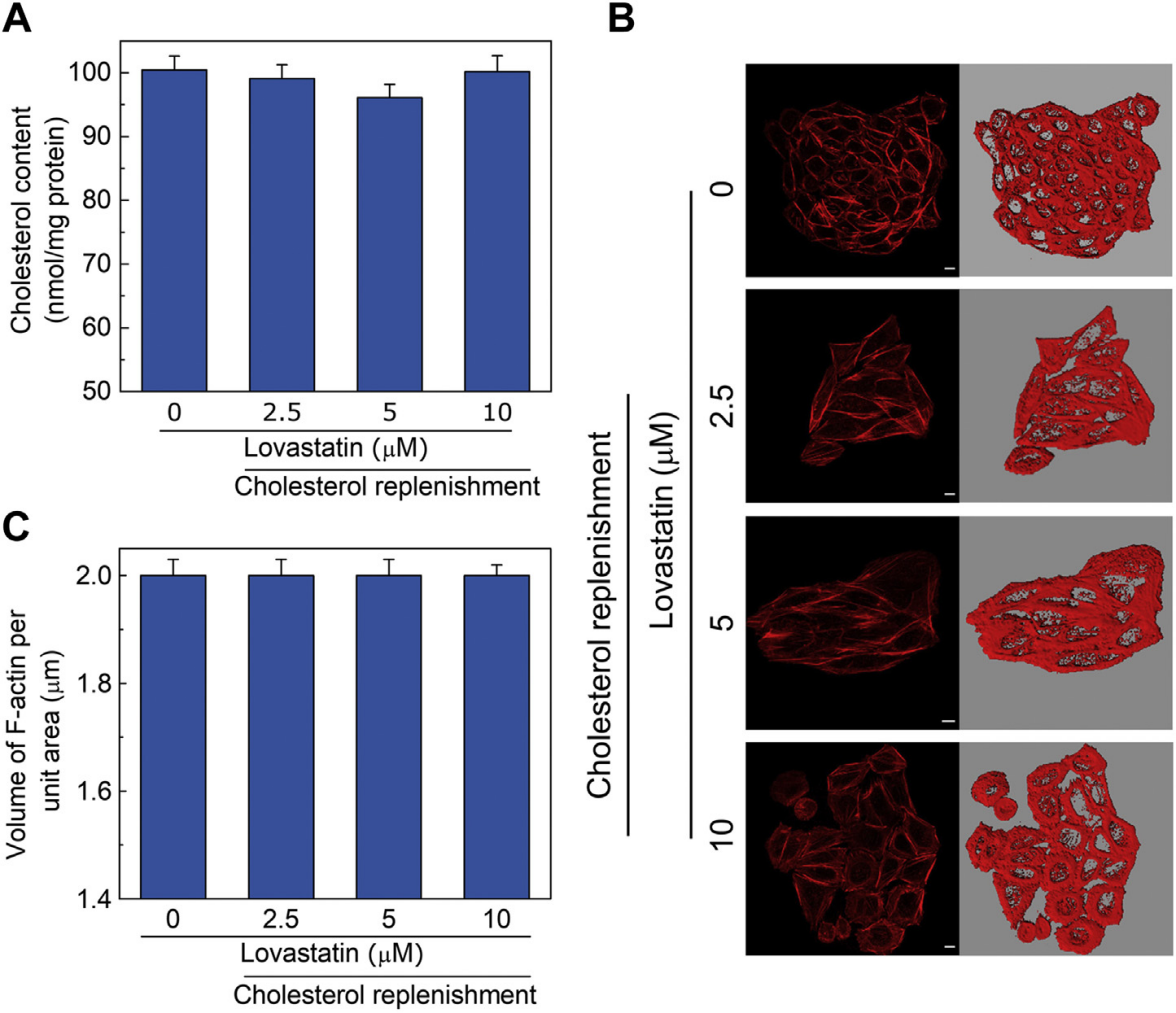
Chronic cholesterol depletion induced by actin polymerization is reversible in nature. Cholesterol was metabolically replenished in lovastatin-treated CHO-K1 cells by further incubating cells in complete culture medium without lovastatin. A: Cholesterol content in CHO-K1 cells under control and cholesterol-replenished (subsequent to treatment with increasing concentrations of lovastatin) conditions. Values are expressed as absolute cholesterol content in cell membranes and are normalized to protein content. Data represent means ± SEM of at least three independent experiments. B: Representative confocal micrographs showing organization of F-actin in control and cholesterol-replenished (subsequent to treatment with increasing concentrations of lovastatin) cells. The scale bars represent 10 μm. Values obtained upon quantitation of F-actin (as described in Fig. 1) in control and cholesterol-replenished (subsequent to treatment with increasing concentrations of lovastatin) cells are shown in panel (C). Data represent means ± SEM of ∼30 different fields from at least three independent experiments. See Materials and methods for more details.

To test the reversibility of the effect of chronic cholesterol depletion on the dynamics of actin cytoskeleton, we carried out replenishment of cholesterol in lovastatin-treated CHO-K1 cells. For this, we metabolically replenished cholesterol in cholesterol-depleted cells by washing off the media containing lovastatin and incubated these cells further in complete growth medium. Figure 4A shows that culturing cells in complete growth medium replenishes cholesterol to normal levels. We subsequently monitored the level of F-actin under these conditions. Figure 4B shows representative confocal micrographs, highlighting the organization of F-actin in control and cholesterol-replenished (subsequent to treatment with increasing concentrations of lovastatin) cells. The F-actin content under these conditions is shown in Fig. 4C. As shown in the figure, upon cholesterol replenishment, the F-actin content was restored to levels observed for untreated cells across all lovastatin concentrations. Interestingly, the reduction in F-actin content upon cholesterol replenishment was accompanied by a reversal in cellular morphology (supplemental Fig. S10). These results show that the F-actin level in cholesterol-depleted cells is restored back to physiological levels upon replenishment of cholesterol, thereby highlighting the reversible nature of actin cytoskeleton polymerization.

### Acute cholesterol depletion has a differential effect on actin polymerization

**Fig. 5:**
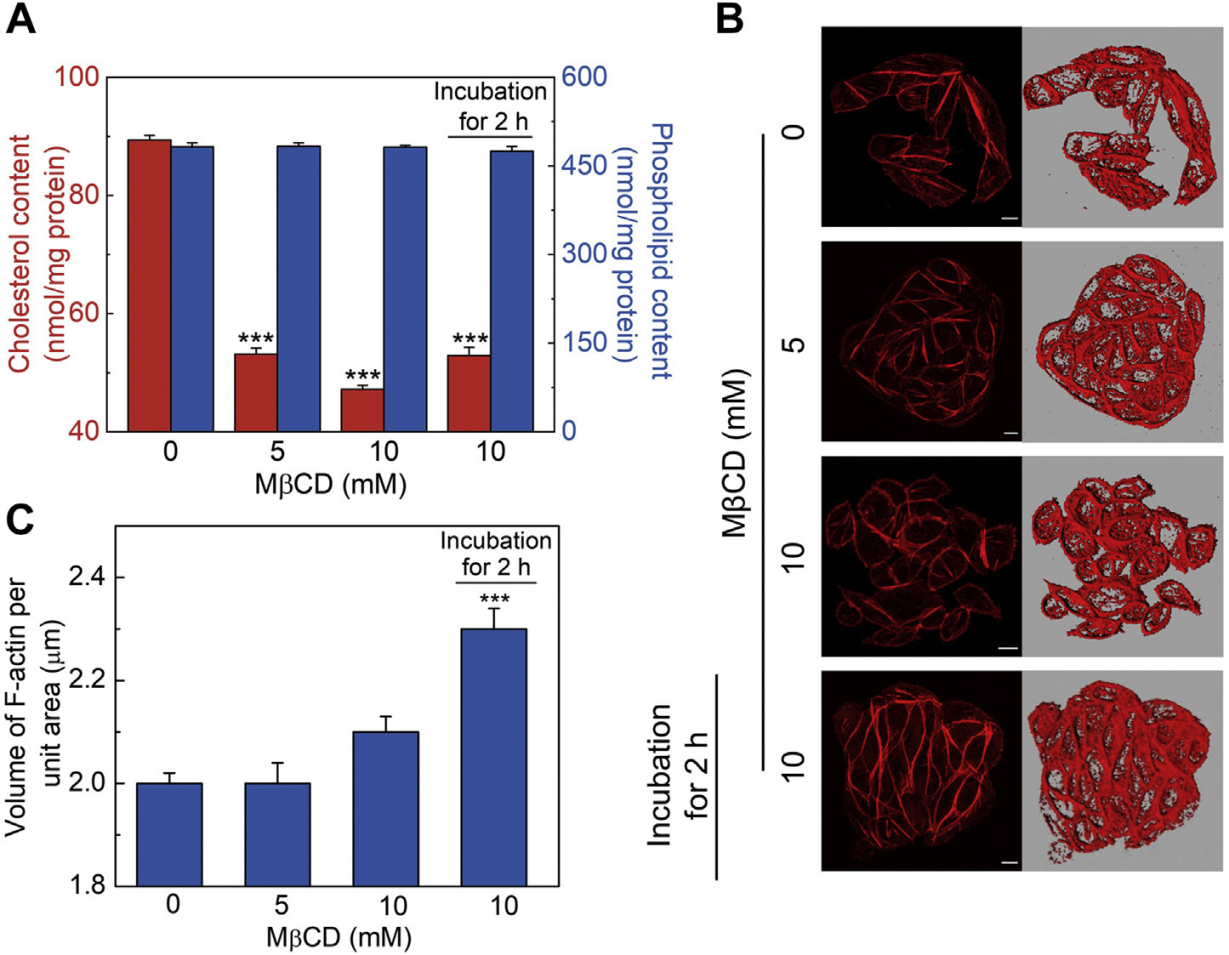
Acute cholesterol depletion shows a differential effect on actin polymerization. CHO-K1 cells were acutely depleted of membrane cholesterol using MβCD. To explore the kinetics of the reorganization of actin cytoskeleton upon acute cholesterol depletion, cells treated with 10 mM MβCD were washed with PBS and incubated in serum-free culture medium for 2 h. A: Effect of acute cholesterol depletion on lipid composition of cell membranes. Cholesterol and phospholipid contents (red and blue bars, respectively) are expressed as absolute levels of the respective lipids in cell membranes and are normalized to membrane protein content. Data represent means ± SEM of at least three independent experiments (∗∗∗ corresponds to a significant difference [*P* < 0.001] in membrane cholesterol content in cholesterol-depleted cells relative to untreated cells). The change in phospholipid content is negligible under identical conditions, thereby indicating the specificity of MβCD in extracting membrane cholesterol. B: Representative confocal micrographs showing organization of F-actin in control and under conditions of acute cholesterol depletion. Panels on the right represent the iso-surfaces, generated as described in Fig. 1. The scale bars represent 10 μm. Values obtained upon quantitation of F-actin in control and cholesterol-depleted cells are shown in panel (C). Data represent means ± SEM of ∼30 different fields from at least three independent experiments (∗∗∗ corresponds to a significant difference [*P* < 0.001] in F-actin content in cholesterol-depleted cells relative to untreated cells). See Materials and methods for more details.

Next, we carried out quantification of F-actin under conditions of acute (faster time scale) cholesterol depletion. Acute cholesterol depletion is achieved by physical depletion of cholesterol in a relatively short time of treatment using MβCD, a water-soluble carrier that can selectively and efficiently extract cholesterol from membranes by including it in a central nonpolar cavity (78, 79). Membrane cholesterol depletion using MβCD suffers from a number of limitations (78). A major limitation is that cholesterol depletion using MβCD is an acute process due to the relatively short duration (∼minutes) of treatment compared to a physiological scenario where cholesterol-lowering drugs (such as statins) take longer time (hours to days) for cholesterol depletion (80). Nonetheless, cholesterol depletion using MβCD enjoys considerable popularity due to its specificity to membrane cholesterol and minimal side effects. Figure 5A shows cholesterol content in membranes isolated from cholesterol-depleted CHO-K1 cells using MβCD. Upon treatment with increasing concentrations of MβCD for 30 min, we observed a progressive reduction of the membrane cholesterol content (red bars). When cells were treated with 5 mM MβCD, the cholesterol content was reduced by ∼47% of control (untreated) (Fig. 5A). Maximum reduction (∼53%) of cholesterol content of cell membranes was achieved with 10 mM MβCD. We chose the concentration range of MβCD carefully to minimize loss of cell viability during the time course of the experiment (supplemental Fig. S11). Importantly, the change in phospholipid content under identical conditions was negligible even in the presence of 10 mM MβCD (see blue bars in Fig. 5A), therefore suggesting selectivity of MβCD in terms of membrane cholesterol depletion. We subsequently performed F-actin quantification under conditions of acute cholesterol depletion (Fig. 5B, C). Figure 5C shows that the F-actin level remains invariant when membrane cholesterol was depleted acutely using MβCD. This is in contrast with our observation of increased F-actin level upon chronic cholesterol depletion using lovastatin (Fig. 1D). Our results therefore suggest that the cellular F-actin level is more sensitive to membrane cholesterol content when cholesterol is depleted in a chronic fashion. These results indicate that the actual process by which cholesterol depletion is carried out (acute vs. chronic) determines the dynamics of the actin cytoskeleton rather than mere cholesterol content.

In order to test whether the observed invariance in F-actin levels upon acute cholesterol depletion was due to the shorter time scale associated with the depletion process, we incubated cholesterol-depleted cells further in MβCD-free medium and carried out F-actin quantification. Interestingly, we observed a significant increase in F-actin levels when cholesterol-depleted cells were incubated in serum-free growth medium for 2 h (Fig. 5B, C). Importantly, the reduced membrane cholesterol levels persisted (at ∼53% of that in untreated cells) under these conditions (Fig. 5A), indicating that the increase in F-actin is not due to the de novo cholesterol biosynthesis in these cells. These results suggest that the kinetics of cholesterol depletion process and subsequent redistribution/organization of cholesterol is crucial in regulating the crosstalk between membrane cholesterol and the actin cytoskeleton.

### Mechanism of actin polymerization upon cholesterol depletion

**Fig. 6:**
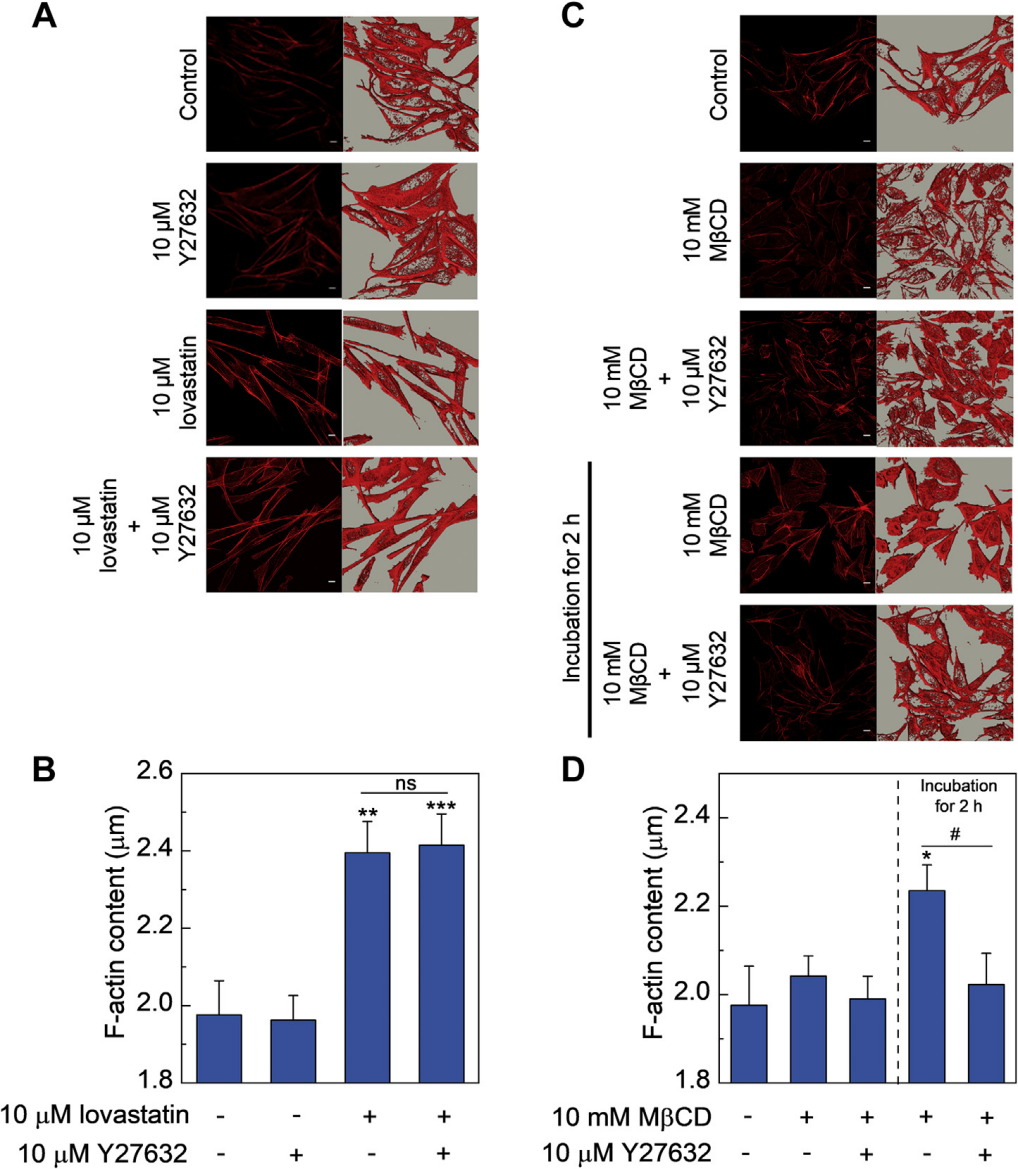
Role of RhoA activation in polymerization of actin cytoskeleton. A: Representative confocal micrographs showing organization of F-actin in control and cells treated 10 μM lovastatin for 48 h in the absence or presence of 10 μM ROCK inhibitor Y27632. F-actin was labeled and visualized as described in Fig. 1. The scale bars represent 10 μm. Values obtained upon quantitation of F-actin in control and cholesterol-depleted cells are shown in panel (B). To quantify the F-actin content, the cellular volume enclosed by the iso-surface was normalized to the projected area of cells. Data represent means ± SEM of ∼20 different fields from three independent experiments (∗∗ and ∗∗∗ correspond to a significant [*P* < 0.01 and *P* < 0.001] difference in F-actin content in lovastatin-treated cells and lovastatin-treated cells in the presence of Y27632 relative to control cells). The lack of significant difference between F-actin content of lovastatin-treated cells relative to lovastatin-treated cells in the presence of Y27632 is denoted by ns. C: Representative confocal micrographs showing organization of F-actin in control cells and cells treated with 10 mM MβCD for 30 min in the absence or presence of 10 μM Y27632. Cells treated with 10 mM MβCD that were washed with PBS and incubated in serum-free culture medium for 2 h in the absence or presence of 10 μM Y27632 are also shown. F-actin was labeled and visualized as described in Fig. 1. The scale bars represent 10 μm. Values obtained upon quantitation of F-actin in control and cholesterol-depleted cells are shown in panel (D). To quantify the F-actin content, the cellular volume enclosed by the iso-surface was normalized to the projected area of cells. Data represent means ± SEM of ∼20 different fields from three independent experiments (∗ corresponds to a significant [*P* < 0.05] difference in F-actin content in MβCD-treated cells cultured for 2 h post cholesterol depletion relative to control cells; # corresponds to a significant [*P* < 0.05] difference in F-actin content in MβCD-treated cells cultured for 2 h post cholesterol depletion in the absence or presence of Y27632). See Materials and methods for more details.

Reduction in membrane cholesterol was shown to increase membrane stiffness via an actin cytoskeleton-dependent mechanism (81, 82). Actin cytoskeleton assembly is controlled by Rho family GTPases, specifically RhoA (83). It has been previously suggested that acute cholesterol depletion using MβCD leads to Src kinase-mediated RhoA GTPase activation in a time-dependent manner (84). Downstream of RhoA is ROCK, a serine/threonine kinase which is activated by RhoA binding, leading to prominent stress fiber formation (83, 84, 85, 86). We therefore tested the possible involvement of ROCK, by using a specific ROCK inhibitor Y27632 (87, 88), and explored its effect on cholesterol depletion-mediated actin polymerization under both acute and chronic cholesterol-depleted conditions. The rationale behind our approach was that if activation of RhoA is involved in polymerization of actin cytoskeleton upon cholesterol depletion, an inhibitor of the RhoA pathway should prevent actin polymerization under these conditions. As shown in Fig. 6 and discussed earlier (Fig. 1), we observed a significant increase in F-actin content under 10 μM lovastatin-treated condition. Notably, there was no significant change in F-actin content when cells were treated with lovastatin in the presence of 10 μM Y27632 as compared to lovastatin alone (Fig. 6A, B). Strikingly, in case of MβCD-treated cells cultured for 2 h post cholesterol depletion, treatment with Y27632 prevented cholesterol depletion induced actin polymerization (Fig. 6C, D). These results highlight the key molecular differences in cholesterol-dependent actin cytoskeleton reorganization under acute and chronic depletion conditions.

**Fig. 7:**
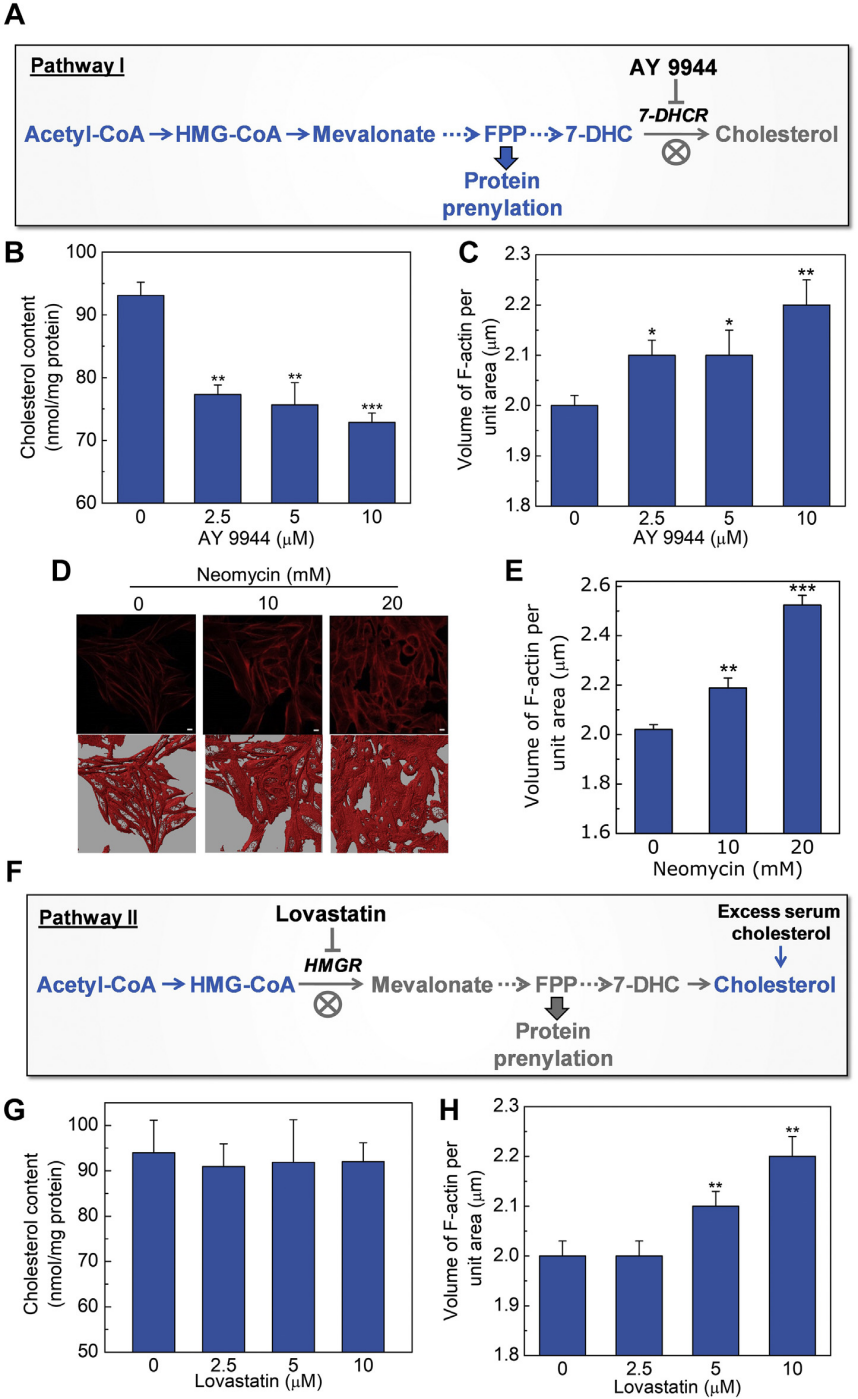
Role of plasma membrane PI(4,5)P_2_ and protein prenylation in actin dynamics. A: To explore the specific contribution of PI(4,5)P_2_-mediated regulation on F-actin dynamics, we chose a distal inhibitor of the cholesterol biosynthesis pathway that minimally affects isoprenoid biosynthesis (e.g., farnesyl pyrophosphate, an important lipid attachment for the post-translational modification of a variety of small GTPase proteins that are key regulators of the actin cytoskeleton). For this, we utilized AY 9944, which is a specific metabolic inhibitor of 7-dehydrocholesterol reductase (7-DHCR), the last enzyme in the Kandutsch-Russell pathway of cholesterol biosynthesis, that catalyzes the conversion of 7-dehydrocholesterol (7-DHC) to cholesterol. The two-way color scheme (inhibition [gray] and operational [blue]) is suggestive of modulation of specific steps of the cholesterol biosynthesis pathway upon AY 9944 treatment. The dotted arrows represent multiple intermediate steps. B: Membrane cholesterol content of CHO-K1 cells treated with increasing concentrations of AY 9944. Sterols were extracted and quantitated using GC-MS analysis and identified with pure standards. Values are expressed as absolute cholesterol content in cell membranes and are normalized to protein content. Data represent means ± SEM of three independent experiments (∗∗ and ∗∗∗ correspond to a significant difference [*P* < 0.01 and *P* < 0.001, respectively] in membrane cholesterol content in AY 9944-treated cells relative to untreated cells). C: Values obtained upon quantitation of F-actin (as described in Fig. 1) in control and AY 9944-treated cells. Data represent means ± SEM of ∼35 different fields from at least three independent experiments (∗ and ∗∗ correspond to significant differences [*P* < 0.05 and *P* < 0.01, respectively] in F-actin content in AY 9944-treated cells relative to untreated cells). D: Effect of sequestering PI(4,5)P_2_ on F-actin dynamics. Representative confocal micrographs showing organization of F-actin in control and cells treated with increasing concentrations of neomycin. Increase in F-actin filaments can be observed upon treatment with increasing concentrations of neomycin. Panels on the right represent the iso-surfaces corresponding to MIPs shown in left panels. The scale bars represent 10 μm. Values obtained upon quantitation of F-actin in control and cells treated with neomycin are shown in panel (E). Data represent means ± SEM of ∼10 different fields from three independent experiments (∗∗ and ∗∗∗ correspond to significant [*P* < 0.01 and *P* < 0.001, respectively] differences in F-actin content in neomycin-treated cells relative to control cells). F: To explore the specific contribution of protein prenylation (an important post-translational modification of a variety of small GTPase proteins that are key regulators of the actin cytoskeleton) on F-actin dynamics, we used lovastatin as a proximal inhibitor of the cholesterol biosynthesis pathway. Lovastatin is a specific metabolic inhibitor of HMG-coA reductase (HMGR), the rate-limiting enzyme upstream of isoprenoid intermediates in the cholesterol biosynthesis pathway. In order to replenish membrane cholesterol in cells during lovastatin treatment, cells were grown in the presence of excess serum, which effectively replenishes cholesterol metabolically. The color scheme is same as in panel A. G: Membrane cholesterol content of CHO-K1 cells treated with increasing concentrations of lovastatin in media containing 20% (v/v) serum. Data represent means ± SEM of three independent experiments. Values are expressed as absolute cholesterol content in cell membranes and are normalized to protein content. H: Values obtained upon quantitation of F-actin (as described in Fig. 1) in control and lovastatin-treated cells. Data represent means ± SEM of ∼20 different fields from at least three independent experiments (∗∗ corresponds to a significant difference [*P* < 0.01] in F-actin content in lovastatin-treated cells relative to untreated cells). See Materials and methods for more details.

What is the molecular mechanism that regulates the crosstalk between membrane cholesterol and actin cytoskeleton under chronic cholesterol depletion? To explore this aspect, we utilized inhibitors that target different steps in the cholesterol biosynthetic pathway (see inset in Figs. 2A and 7A). Statin is a proximal inhibitor of the multistep cholesterol biosynthesis pathway and blocks the biosynthesis of isoprenoid intermediates such as farnesyl pyrophosphate and geranylgeranyl pyrophosphate, which serve as important lipid attachments for intracellular trafficking and functional activation of a variety of membrane-associated proteins (89). These proteins include small GTP binding proteins belonging to the family of Rho GTPases that are the master regulators of actin cytoskeleton organization and dynamics (29, 90). It has previously been shown that chronic cholesterol depletion by lovastatin leads to accumulation of nonisoprenylated Rho and Rac GTPases in the cytoplasm (91) that could result in altered cytoskeletal dynamics. In addition, depletion of membrane cholesterol leads to loss and subsequent redistribution of plasma membrane PI(4,5)P_2_ (16), one of the most well-studied phospholipids that mediate the crosstalk between the plasma membrane and cytoskeleton network via binding to cytoskeletal proteins and actin nucleators (92).

To dissect out the possible contributions from these pathways that regulate cellular cytoskeletal dynamics, we adopted a two-pronged approach. First, to explore the possible role of PI(4,5)P_2_ in actin polymerization, we chose a distal inhibitor of the cholesterol biosynthetic pathway that does not perturb isoprenoid biosynthesis. For this, we utilized AY 9944, which is a specific metabolic inhibitor of 7-dehydrocholesterol reductase (47), the last enzyme that catalyzes the conversion of 7-dehydrocholesterol to cholesterol in the Kandutsch-Russell pathway of cholesterol biosynthesis (Fig. 7A). The membrane cholesterol content of CHO-K1 cells treated with increasing concentrations of AY 9944 is shown in Fig. 7B. As shown in the figure, treatment with AY 9944 led to significant reduction in membrane cholesterol content with maximum reduction (∼27%) observed upon treatment with 10 μM AY 9944. Importantly, cell viability was not compromised even in the presence of the highest concentration of AY 9944 (supplemental Fig. S12). We observed a significant increase in F-actin content (∼10% with highest concentration of AY 9944, see Fig. 7C) upon treatment with increasing concentrations of AY 9944. To further validate these observations, we sequestered plasma membrane PI(4,5)P_2_ (without affecting its level by depletion of membrane cholesterol) and measured F-actin content (Fig. 7D, E). If regulation of the actin cytoskeleton dynamics depends on the availability of PI(4,5)P_2_ in the plasma membrane, then we would expect that sequestering PI(4,5)P_2_ should have effects similar to cholesterol depletion. To test this hypothesis, we cultured CHO-K1 cells in the presence of neomycin, an aminoglycoside antibiotic that specifically interacts with PI(4,5)P_2_ in the membrane (93), thereby effectively reducing the ability of PI(4,5)P_2_ to interact with and exert its effects on proteins involved in cytoskeletal dynamics. Figure 7D shows that incubating cells with increasing concentrations of neomycin led to polymerization of F-actin and subsequent increase in F-actin content (Fig. 7E).

**Fig. 8:**
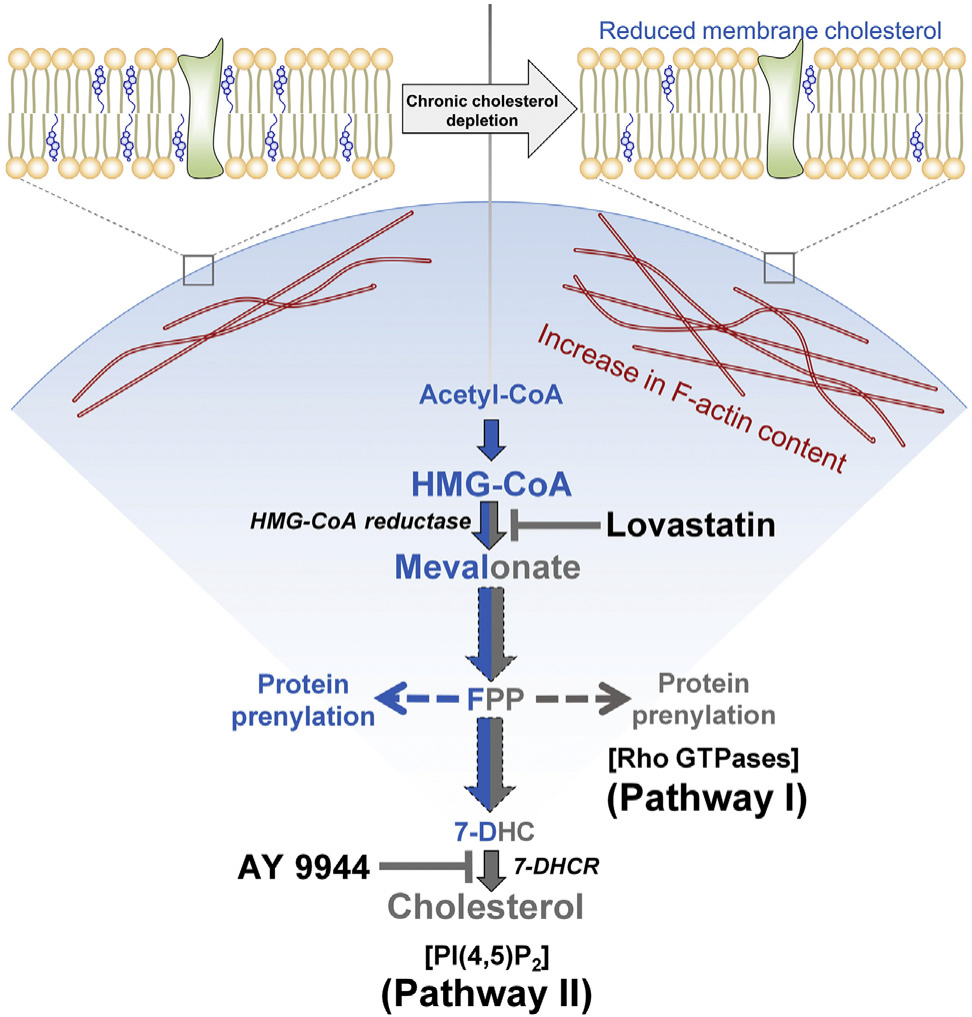
Dual mechanism of regulation of cellular actin polymerization through the cholesterol biosynthetic pathway. A significant increase in F-actin content associated with reduction in membrane cholesterol was observed under chronic cholesterol depleted conditions. Cholesterol depletion utilizing lovastatin (a proximal inhibitor of the cholesterol biosynthesis pathway) and AY 9944 (a distal inhibitor of the cholesterol biosynthesis pathway) suggested specific contributions of protein prenylation (pathway I) and PI(4,5)P_2_ (pathway II) underlying the altered F-actin dynamics. Whereas protein prenylation is an important post-translational modification of a variety of small GTPase proteins that are key regulators of the actin cytoskeleton, PI(4,5)P_2_ is a phospholipid that mediates the crosstalk between the plasma membrane and cytoskeleton network via binding to cytoskeletal proteins and actin nucleators. Lovastatin is a specific metabolic inhibitor of HMG-CoA reductase (HMGR), the rate-limiting enzyme upstream of isoprenoid intermediates in the cholesterol biosynthesis pathway. On the other hand, AY 9944 is a specific metabolic inhibitor of 7-dehydrocholesterol reductase (7-DHCR), the last enzyme in the Kandutsch-Russell pathway of cholesterol biosynthesis, that catalyzes the conversion of 7-dehydrocholesterol (7-DHC) to cholesterol. The two-way color scheme (inhibition [gray] and operational [blue]) is suggestive of modulation of specific steps upon lovastatin (right half of the pathway) and AY 9944 (left half of the pathway). The dotted arrows represent multiple biosynthesis steps. See text for more details.

In an alternate approach, to explore the specific contribution of protein prenylation on F-actin dynamics, we used lovastatin as a proximal inhibitor of the mevalonate pathway (Fig. 7F). In addition, we replenished membrane cholesterol during lovastatin treatment by growing cells in the presence of excess serum, which effectively metabolically replenishes cholesterol. This ensures an optimal level of membrane cholesterol similar to control cells even when lovastatin is present in the culture medium. We observed no significant change in membrane cholesterol level when cells were grown in the presence of medium containing lovastatin and supplemented with 20% (v/v) serum (Fig. 7G). This suggests that additional serum in the cell culture medium was sufficient to provide enough cholesterol for maintenance of an optimal level of membrane cholesterol during lovastatin treatment. Interestingly, although there was no difference in membrane cholesterol content in control and lovastatin-treated cells, we observed a significant increase in F-actin content with higher concentrations of lovastatin (see Fig. 7H). We interpret the change in F-actin content under conditions of identical membrane cholesterol due to cholesterol-independent mechanisms, such as lack of protein prenylation. Taken together, these results suggest that the interplay between membrane cholesterol and the actin cytoskeleton could be regulated by synergy of two pathways that are associated with cellular cholesterol biosynthesis (Fig. 8).

## Discussion

Cholesterol is an essential component of cellular membranes of all higher eukaryotes and is crucial in membrane organization, dynamics, function, and sorting (34). In humans, cholesterol is obtained through diet and by de novo synthesis, occurring primarily in the liver, from acetyl-CoA via a long and multistep sterol biosynthesis pathway (35). The contribution of de novo cholesterol biosynthesis versus dietary intake for total body cholesterol is estimated to be ∼70:30 (94). High cholesterol level in blood, also known as hypercholesterolemia, increases the risks of heart diseases and stroke and is estimated to cause ∼2.6 million deaths every year (https://www.who.int/data/gho/indicator-metadata-registry/imr-details/3236). In this context, statins are extensively administered as oral cholesterol-lowering agents and have emerged as one of the best-selling drugs globally. However, besides beneficial effects of cholesterol lowering, use of statins is often complicated due to its adverse side effects. Many of these cholesterol-independent side effects are mediated by inhibition of biosynthesis of isoprenoids, which are post-translationally attached to crucial intracellular signaling molecules.

Membrane cholesterol has emerged as a key modulator of the function of membrane proteins, such as GPCRs (95). Modulation of plasma membrane cholesterol offers a useful tool to address cholesterol-dependent organization and function of membrane proteins. Chronic cholesterol depletion using statins has proved to be a convenient approach that mimics the physiological scenario, in contrast to carriers such as MβCD that induce acute cholesterol depletion. However, similar to side effects observed in vivo, statin treatment in cells often leads to pleiotropic effects in addition to reduction of cholesterol (65). In this context, chronic cholesterol depletion has been shown to suppress lateral mobility of several membrane proteins, possibly due to polymerization of membrane-associated actin cytoskeleton (48, 96). In spite of these important functional correlates, the molecular mechanism underlying the modulation of actin cytoskeleton dynamics in response to chronic cholesterol depletion is not well understood.

In this work, we explored the effect of chronic cholesterol depletion using lovastatin on the dynamics of cellular F-actin network in CHO-K1 cells. For this, we employed a confocal microscopy based approach followed by image reconstruction to quantitatively estimate cellular actin content. We show here that F-actin content significantly increases in response to cholesterol depletion, with a concomitant reduction in G-actin levels. Interestingly, metabolic replenishment of membrane cholesterol resulted in reduction of F-actin content to normal levels. We further showed that the effect of cholesterol depletion on polymerization of actin cytoskeleton is not specific to the chemical nature of statin. In addition, keeping in mind the blood-brain-barrier crossing ability of lipophilic statins (68, 69), we showed that neuronal cells display a similar relationship between membrane cholesterol content and F-actin levels. Such dynamic changes in the actin cytoskeleton network in neurons could have functional implications in desensitization and endocytosis of membrane proteins such as ion channels (97). Notably, unlike chronic cholesterol depletion, our results from acute cholesterol depletion using MβCD showed no significant changes in F-actin content. A major difference between acute and chronic depletion is the kinetics of the two processes associated with reduction of membrane cholesterol (80). Our results therefore suggest that the effect of cholesterol depletion on actin cytoskeleton reorganization is not a fast process. While chronic depletion is a relatively slow process and therefore provides enough time for membrane reorganization, acute depletion is a faster process and reorganization of actin cytoskeleton may not be complete under these conditions. As a result, these two processes lead to different consequences in many cases (such as the organization of caveolae and GPI-anchored proteins, induction of autophagy, and activity of sodium/phosphate cotransporter (80)). We have previously shown that dipolar organization in cholesterol-depleted cells is dependent on the method used to deplete membrane cholesterol (80). Importantly, we recently showed that endocytosis and trafficking of the serotonin_1A_ receptor, a key neurotransmitter receptor in the GPCR family, depends on the method of cholesterol depletion (chronic vs. acute) (98, 99). In this backdrop, our results showing differential dynamics of the actin cytoskeleton as a consequence of acute and chronic cholesterol depletion assume relevance.

Finally, we explored the molecular mechanism underlying the sensitivity of actin cytoskeleton to membrane cholesterol content utilizing multiple inhibitors of cholesterol biosynthesis. Our results showed that actin polymerization in response to membrane cholesterol depletion is regulated by multiple pathways with contributions from prenylated Rho GTPases and availability of membrane PI(4,5)P_2_. Since statins inhibit the multistep cholesterol biosynthesis at an early step, statin treatment leads to reduction of a number of intermediates (such as isoprenoids) in the pathway, in addition to reduction in cholesterol (100). Lack of isoprenylation is known to affect membrane association and functional activation of Rho GTPase family of proteins that act as key modulators of cellular actin network (28, 29). It has previously been shown that cholesterol depletion by lovastatin results in accumulation of nonisoprenylated Rho GTPases in the cytosolic fraction (84, 89, 101, 102). Interestingly, depletion of membrane cholesterol is known to affect the organization of PI(4,5)P_2_ (16), a well-characterized signaling lipid that participates in binding to a range of proteins that regulate the dynamics of actin cytoskeleton (92). Our results therefore address the missing links between reorganization of cytoskeleton in relation to the pathways that are intimately associated with cellular cholesterol biosynthesis.

Notably, although acute cholesterol depletion with MβCD showed no change in F-actin levels after 30 min, there was a significant increase in RhoA-dependent increase in F-actin when cells were cultured for 2 h in a cholesterol-depleted condition. It was previously shown that membrane cholesterol depletion using MβCD resulted in Src kinase-mediated Rho activation and caveolin phosphorylation, which led to formation of stress fibers (84). Interestingly, we observed that lovastatin-induced increase in F-actin was insensitive to ROCK inhibition. This could be due to loss of RhoA activity (103) as a result of its accumulation as a nonisoprenylated form in the cytosolic fraction upon lovastatin treatment (84, 89, 101, 102). However, involvement of other prenylation events regulating the increase in F-actin levels should not be ruled out. In addition, it should be noted that the differences between acute and chronic cholesterol depletion could be due to the nature of reorganization of intracellular versus plasma membrane cholesterol under these treatment conditions (104, 105).

Reorganization of actin cytoskeleton is critical for cellular motility, signal transduction, and endocytosis of membrane proteins. Modulation of F-actin content in response to GPCR activation has emerged as an important molecular player in cellular signaling (22, 106). Interestingly, entry and survival of intracellular pathogens is known to be dependent on the status of actin polymerization in host cells (50). With this background, the results outlined in this work showing increase in F-actin upon membrane cholesterol depletion assume significance. We envision a comprehensive understanding of the crosstalk between actin cytoskeleton reorganization and cholesterol biosynthesis pathway would help in deciphering the functional consequences of this interplay.

Depletion of membrane cholesterol offers a convenient strategy to monitor cholesterol dependence of membrane protein dynamics and function. In this context, statins (best-selling cholesterol-lowering drugs) offer a physiological approach to modulate cellular cholesterol levels in a chronic manner. Utilizing various inhibitors of the cholesterol biosynthetic pathway, we show here that cholesterol depletion results in significant polymerization of the actin cytoskeleton. It is therefore advisable to exercise caution before attributing cholesterol dependence in membrane protein dynamics and function to the mere content of membrane cholesterol. To the best of our knowledge, our results represent one of the first comprehensive reports dissecting the mechanistic basis underlying the interplay between cellular actin level and cholesterol biosynthesis. We believe that the dynamic reorganization of the actin cytoskeleton could represent an important determinant in membrane protein dynamics and signaling in diseases that are due to defects in cholesterol biosynthesis pathways such as Smith-Lemli-Opitz syndrome. On a broader and cautionary perspective, we conclude that in analyses of the modulatory role of the membrane environment on the organization and function of membrane proteins, it is prudent to include the actin cytoskeleton as a crucial player.

## Data availability

All data needed to evaluate the conclusions in the paper are present in the paper and/or the Supplementary Materials .

## Supplemental data

This article contains supplemental data.

## Conflicts of interest

The authors declare that they have no competing interests.
