# Supplementary material for: Chronic cholesterol depletion increases F-actin levels and induces cytoskeletal reorganization via a dual mechanism
Source: J Lipid Res. 2022 Apr 4;63(5):100206. doi: 10.1016/j.jlr.2022.100206 (PMC9096963; doi:10.1016/j.jlr.2022.100206)
Supplement: Supplemental Figures S1–S12 [file mmc1.pdf]

Supplemental data for

Chronic cholesterol depletion increases F-actin levels and induces cytoskeletal reorganization *via* a dual mechanism

Parijat Sarkar, G. Aditya Kumar¶, Sandeep Shrivastava and Amitabha Chattopadhyay*

CSIR-Centre for Cellular and Molecular Biology, Uppal Road, Hyderabad 500 007, India

¶Present address: Department of Pharmacology, University of Michigan Medical School, Ann Arbor, MI 48109, USA

*Address correspondence to Amitabha Chattopadhyay, E-mail: amit@ccmb.res.in, Phone: +91-40-2719-2578

Supplemental Figure S1

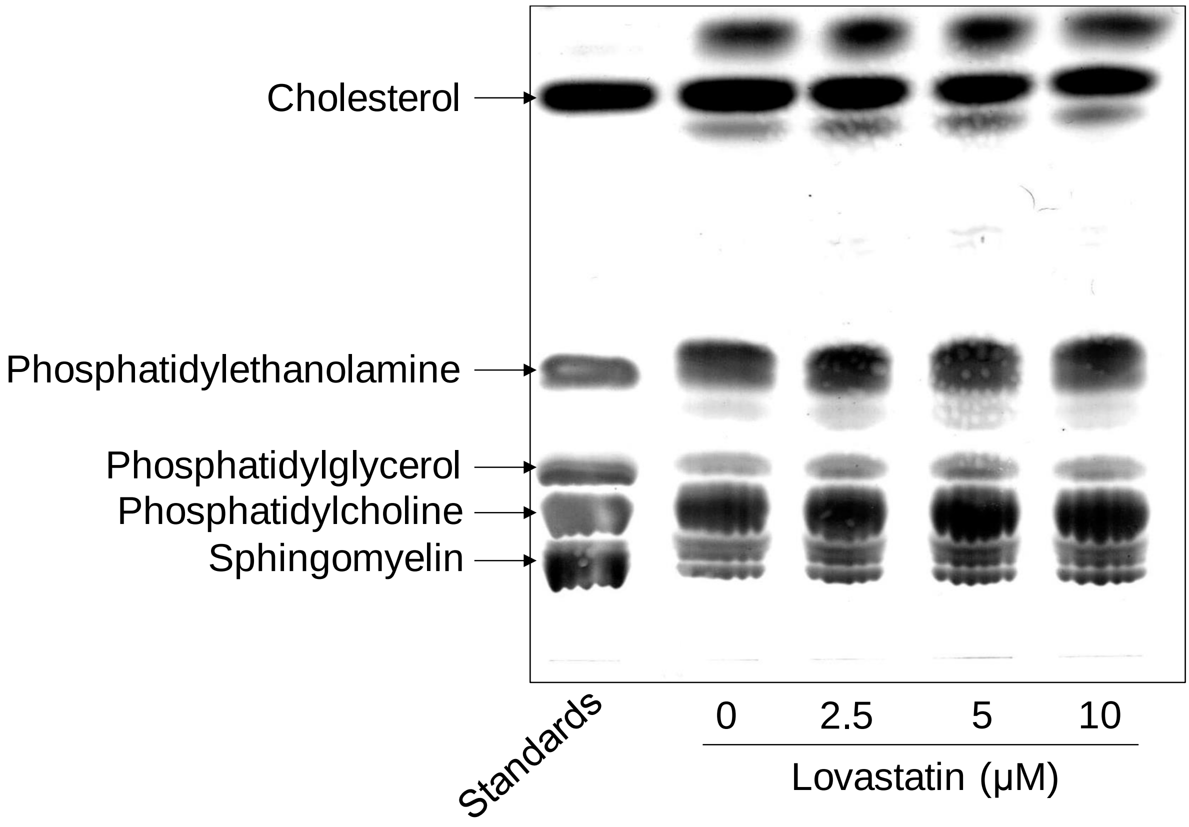

Fig. S1 Thin layer chromatogram of lipid extracts from cell membranes under control and lovastatin-treated conditions. The arrows represent positions of standards. See Materials and Methods for other details.

Supplemental Figure S2

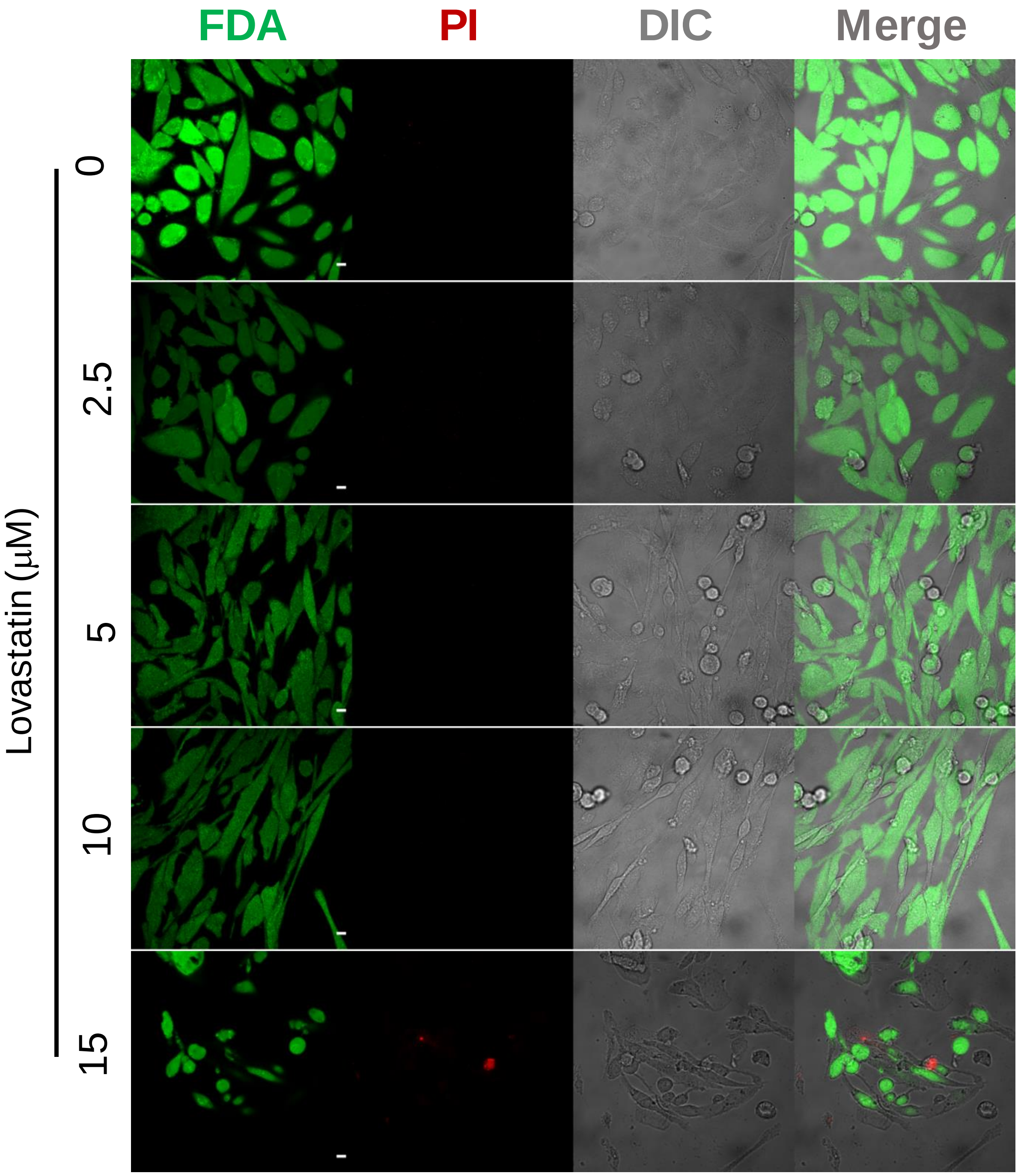

Fig. S2 Viability of CHO-K1 cells upon chronic cholesterol depletion. We utilized a fluorescence-based dual color labeling assay using fluorescein diacetate (FDA) and propidium iodide (PI), which exclusively labels viable cells and dead cells, respectively. FDA is taken up by live cells which convert the non-fluorescent FDA into fluorescent fluorescein. In contrast, PI cannot pass through a viable cell membrane and only enters into dead cells and subsequently labels nucleic acids. Panels from left to right show FDA (green), PI (red) and DIC images of the same field. Merged images are shown in the panel on the extreme right. The scale bars represent 10 μm. See Materials and Methods for more details.

Supplemental Figure S3

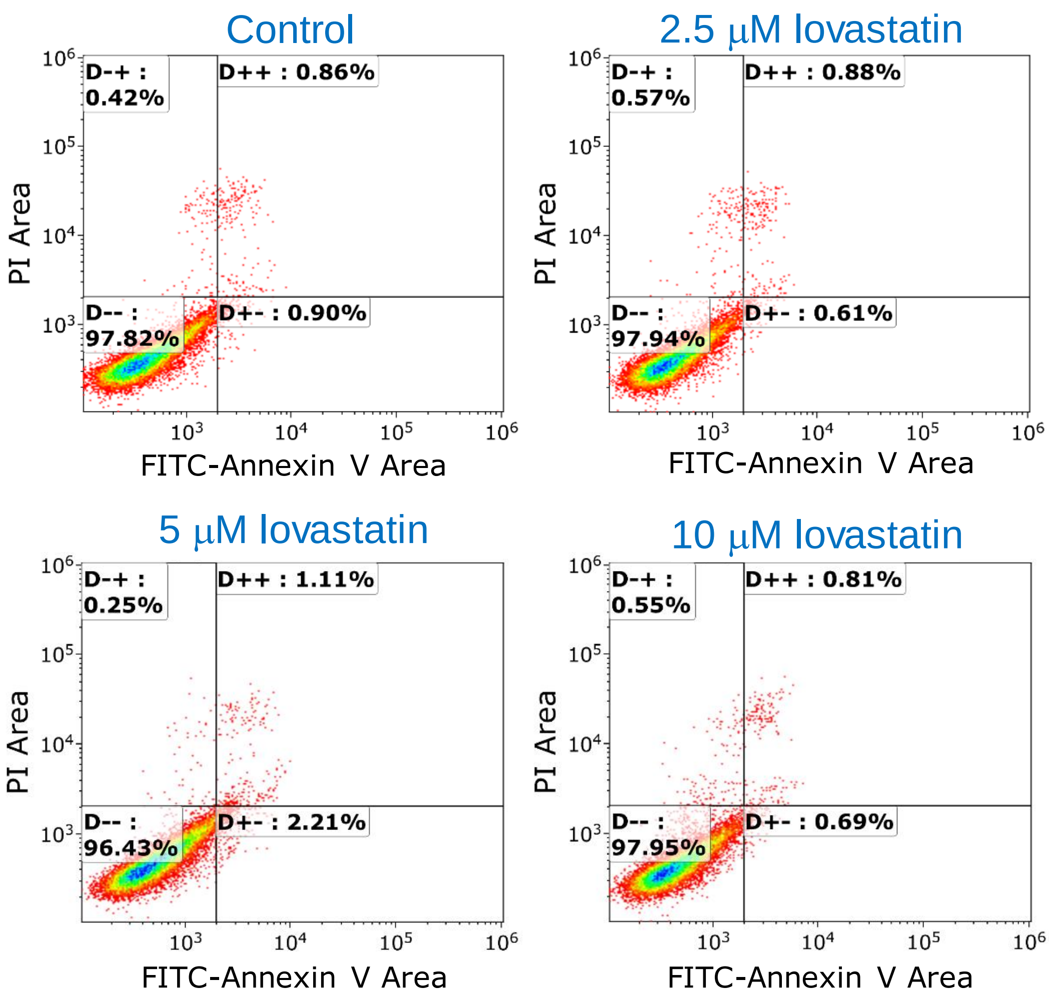

Fig. S3 Effect of chronic cholesterol depletion on apoptosis of CHO-K1 cells. Extent of apoptosis in cells treated with increasing concentrations of lovastatin as indicated over each panel are shown. Apoptosis was measured utilizing a flow cytometry based assay using PI and FITC-Annexin V. The number in each quadrant represents percentage of cells displaying respective phenotype as follows: D--, healthy cells; D+-, cells undergoing early apoptosis; D++, cells undergoing late apoptosis; D-+, cells undergoing necrosis. See Materials and Methods for more details.

Supplemental Figure S4

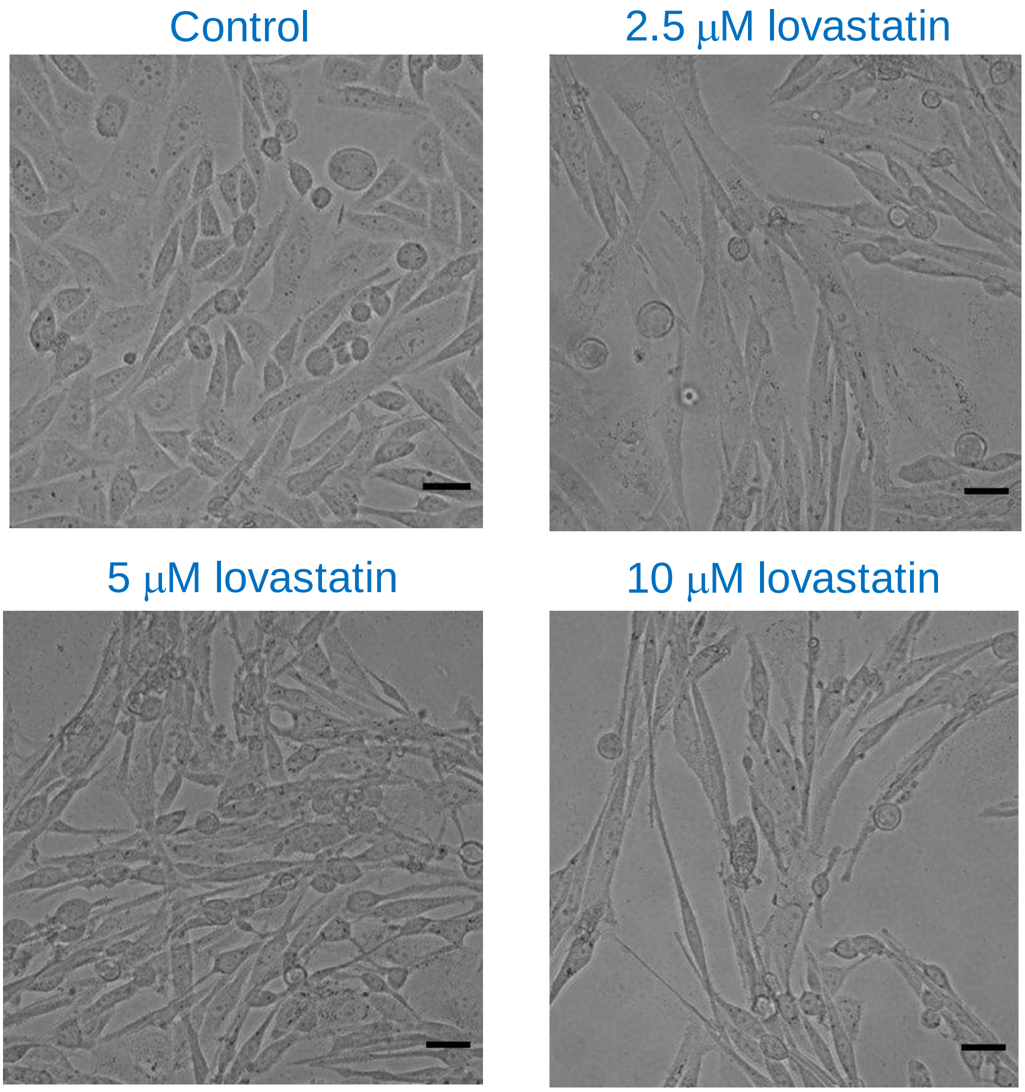

Fig. S4 Morphology of CHO-K1 cells upon treatment with increasing concentrations of lovastatin. Phase-contrast images of cells treated with indicated concentrations of lovastatin for 48 h are shown. The scale bars represent 10 μm.

Supplemental Figure S5

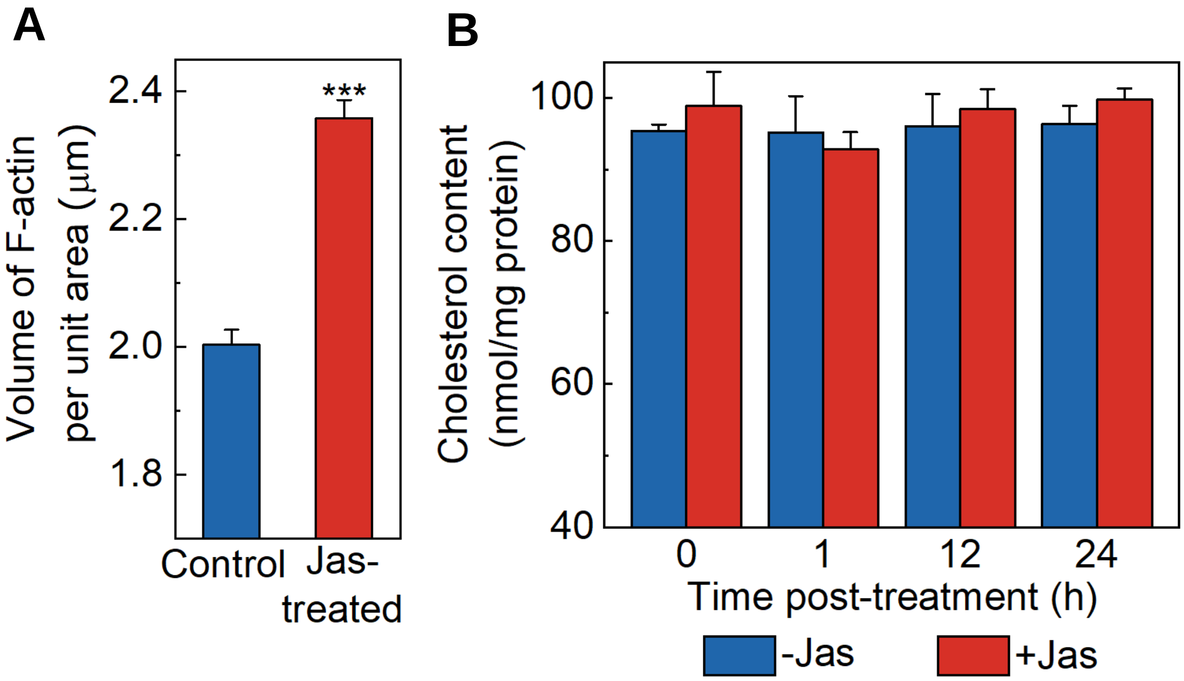

Fig. S5 Actin polymerization has no effect on membrane cholesterol content. (A) Values obtained upon quantitation of F-actin in control (blue) and Jas-treated (red) cells. Data represent means ± SE of ~40 different fields from three independent experiments (*** corresponds to significant ($p < 0.001$) difference in F-actin content in Jas-treated cells relative to control cells). (B) Time-dependent modulation of membrane cholesterol content in cells treated with (red) or without (blue) Jas. Data represent means ± SE of three independent experiments. See Materials and Methods for more details.

Supplemental Figure S6

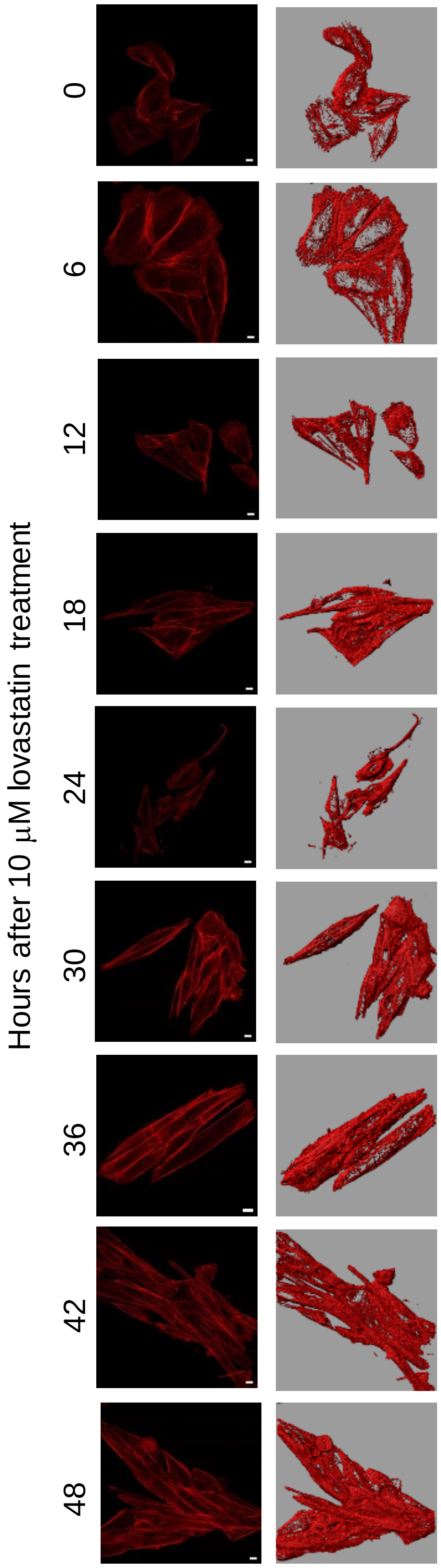

Fig. S6 Time course of actin polymerization in response to chronic cholesterol depletion. CHO-K1 cells treated with 10 μM lovastatin were fixed at specified time intervals and the F-actin was labeled and visualized as described in Fig. 1. Panels on left represent maximum intensity projection (MIP) of confocal micrographs of cells. The corresponding panels on the right represent the iso-surfaces (defined as voxel contours of equal fluorescence intensity) generated from the *z*-sections corresponding to MIPs shown in left panels. The scale bars represent 10 μm. See Materials and Methods for more details.

Supplemental Figure S7

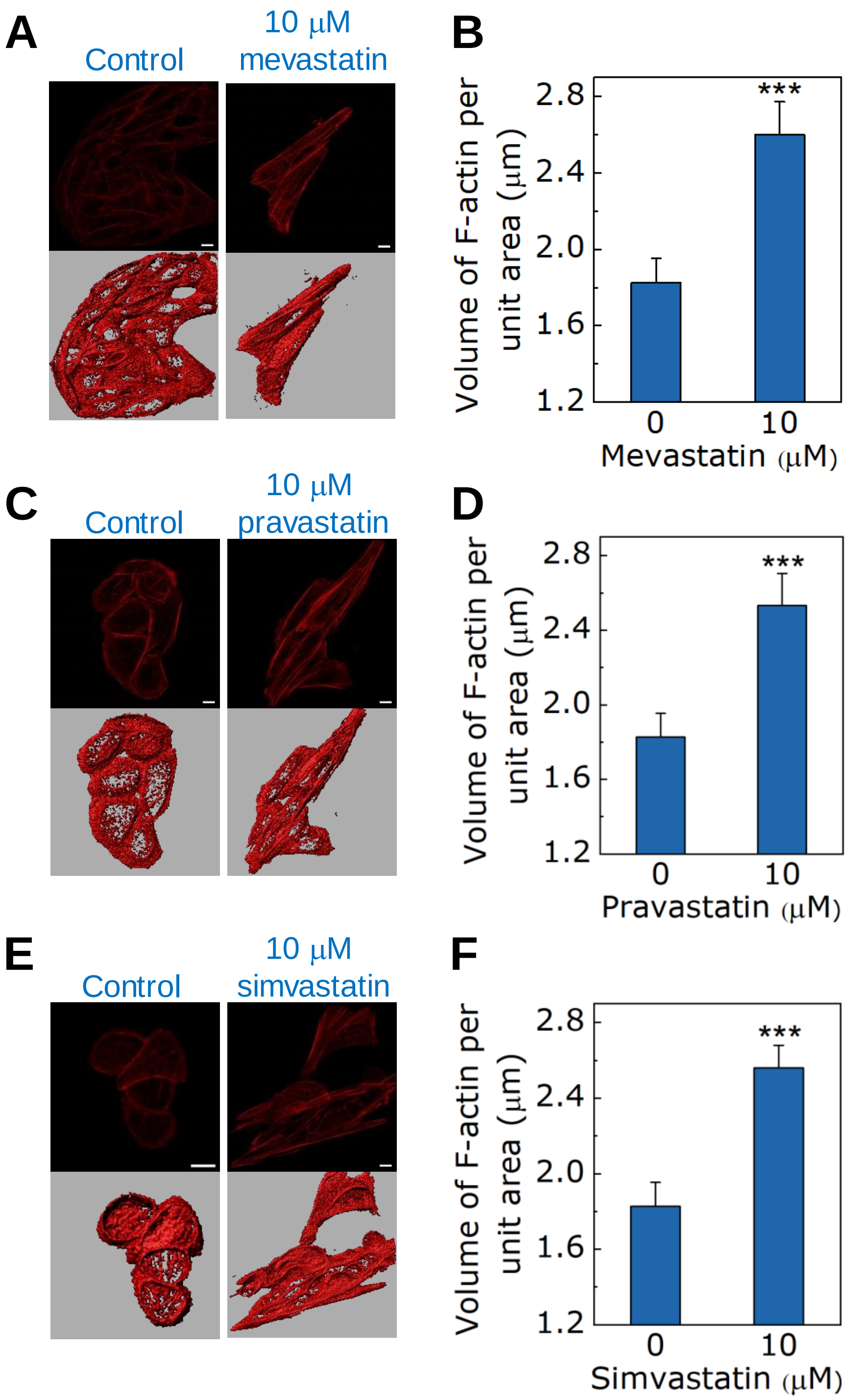

Fig. S7 Actin polymerization upon chronic cholesterol depletion is not specific to a particular statin. Panels (A), (C) and (E) represent confocal micrographs of CHO-K1 cells showing organization of F-actin under mevastatin-, pravastatin- and simvastatin-treated conditions, respectively. F-actin was quantified as described in Fig. 1. The scale bars represent 10 μm. Values obtained upon quantitation of F-actin in mevastatin-, pravastatin- and simvastatin-treated conditions are shown in panels (B), (D) and (F), respectively. Data represent means ±

SE of ~10 different fields from three independent experiments (*** corresponds to significant (p < 0.001) difference in F-actin content in statin-treated cells relative to control cells). See Materials and Methods for more details.

Supplemental Figure S8

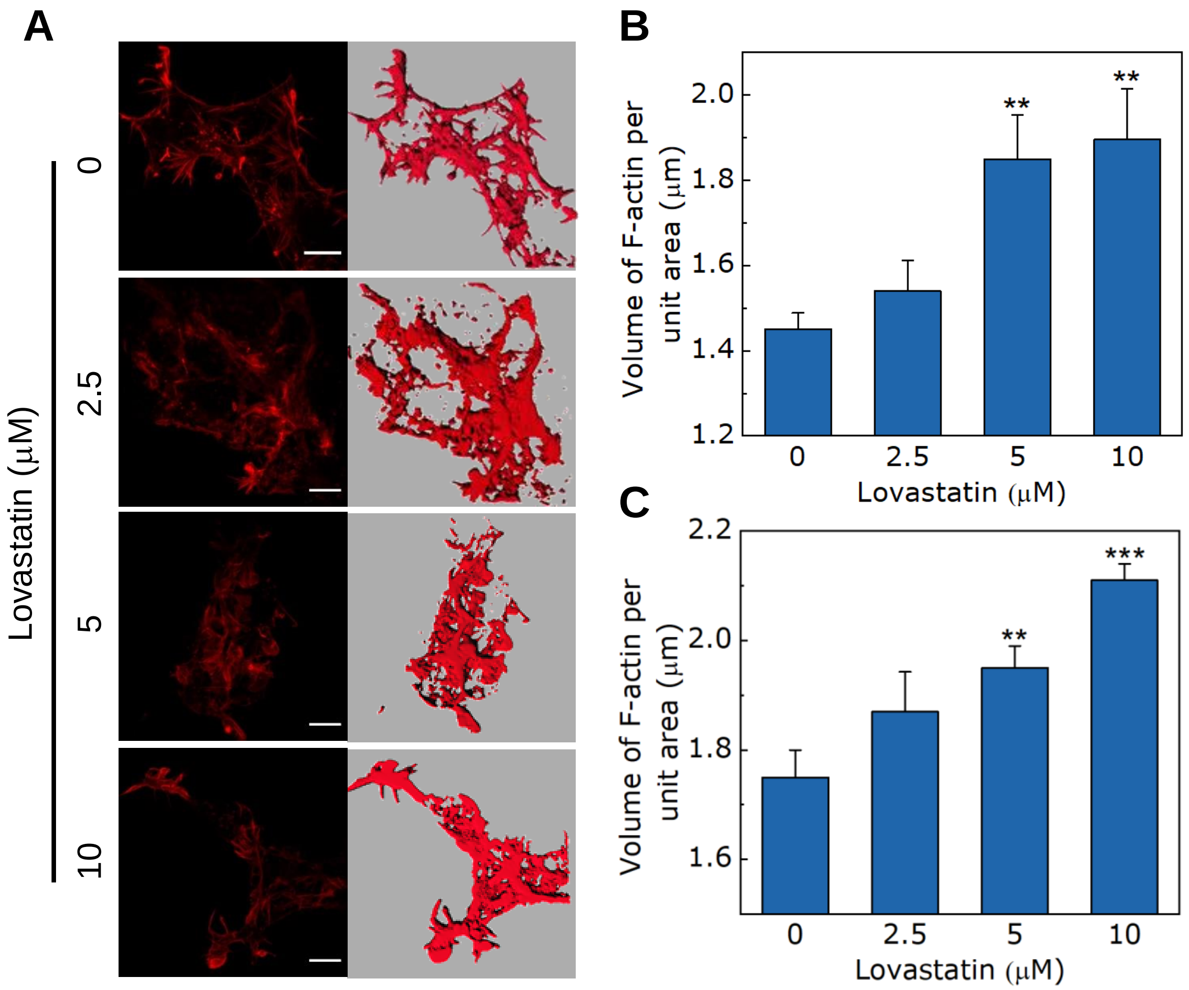

Fig. S8 Actin polymerization in response to chronic cholesterol depletion in neuronal cells. (A) Representative confocal micrographs of HN2 cells showing organization of F-actin in control and lovastatin-treated condition. F-actin was labeled with Alexa Fluor 546 conjugated phalloidin. The maximum intensity projections (MIPs) of 15 *z*-sections from the base of the coverslip (~4.8 μm from the base into the cell) are shown in left panels. The corresponding panels on the right represent iso-surfaces (defined as voxel contours of equal fluorescence intensity) generated from the *z*-sections corresponding to MIPs shown in left panels. The scale bars represent 10 μm. Values obtained upon quantitation of F-actin in control and cholesterol-depleted HN2 cells are shown in panel (B). Data represent means ± SE of ~10 different fields from three independent experiments (** corresponds to significant ($p < 0.01$) difference in F-actin content in cholesterol-depleted cells relative to control cells). (C) Values obtained upon quantitation of F-actin in control and cholesterol-depleted Neuro2a cells. Data represent means ± SE of ~10 different fields from three independent experiments (** and *** corresponds to significant ($p < 0.01$ and $p < 0.001$, respectively) difference in F-actin content in cholesterol-depleted cells relative to control cells). See Materials and Methods for more details.

Supplemental Figure S9

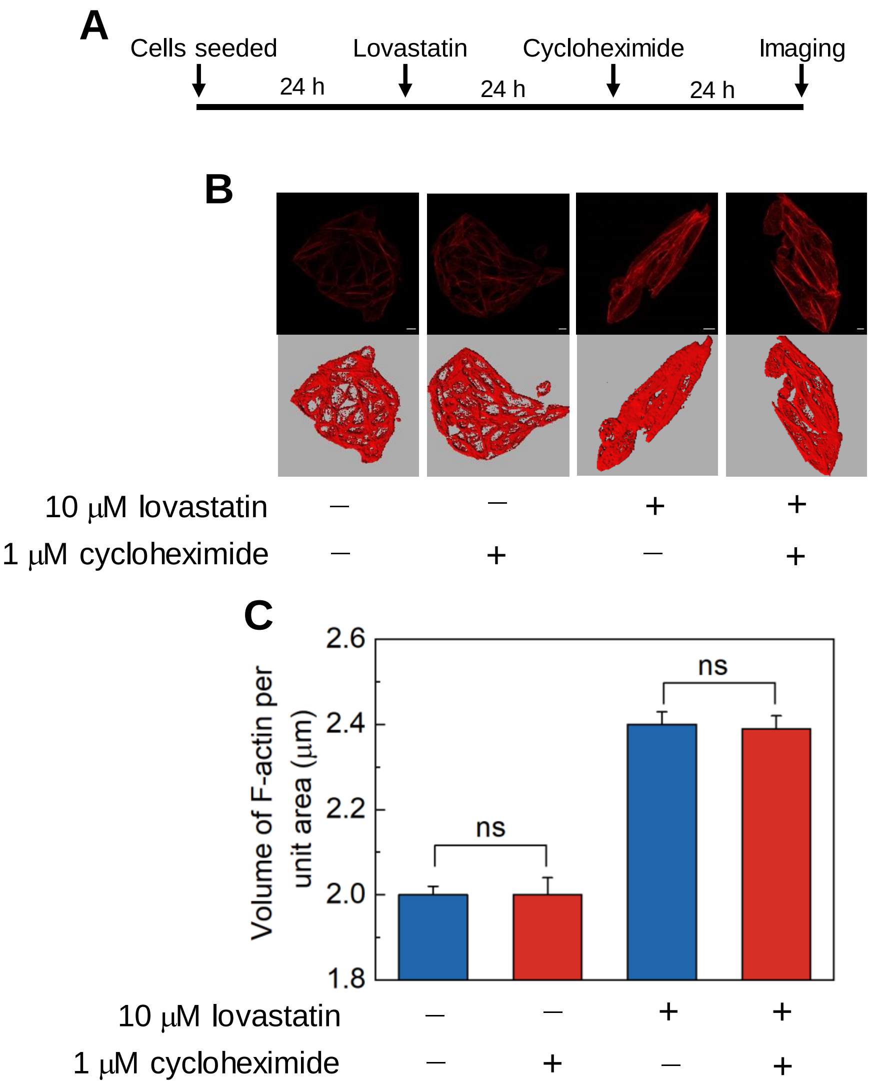

Fig. S9 Actin polymerization upon chronic cholesterol depletion does not require newly synthesized proteins. (A) A schematic of the treatment strategy. CHO-K1 cells grown for 24 h was treated with lovastatin for 48 h. During the last 24 h of lovastatin treatment, the media was supplemented with cycloheximide to block protein synthesis. (B) Representative confocal micrographs of cells showing organization of F-actin cytoskeleton in control and lovastatin-treated condition in the absence or presence of cycloheximide. F-actin was labeled with Alexa Fluor 546 conjugated phalloidin. The maximum intensity projections (MIPs) are shown in top panels. The bottom panels represent the iso-surfaces corresponding to MIPs shown in top panels. The scale bars represent 10 μm. Values obtained upon quantitation of F-actin in control and cholesterol-depleted cells in the presence (red bars) and absence (blue bars) of cycloheximide are shown in panel (C). Data represent means ± SE of at least 25 different fields from three independent experiments. The lack of significant difference between F-actin

content of cycloheximide-treated cells relative to untreated cells is denoted by ns. See Materials and Methods for more details.

Supplemental Figure S10

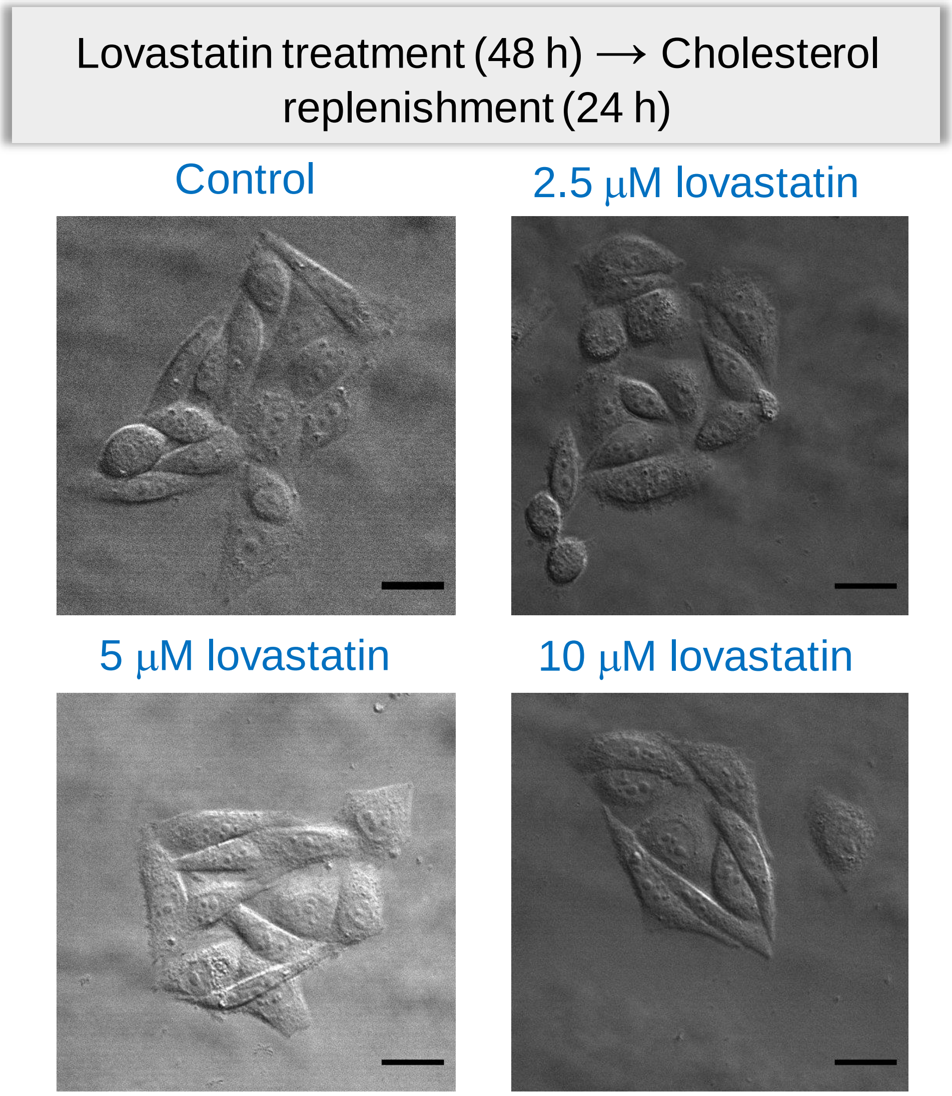

Fig. S10 Morphology of CHO-K1 cells upon cholesterol replenishment. Cholesterol was metabolically replenished in lovastatin-treated CHO-K1 cells by further incubating cells for 24 h in complete DMEM/F-12 medium without lovastatin. DIC images of cells treated with indicated concentrations of lovastatin for 48 h prior to lovastatin withdrawal are shown. The scale bars represent 10 μm.

Supplemental Figure S11

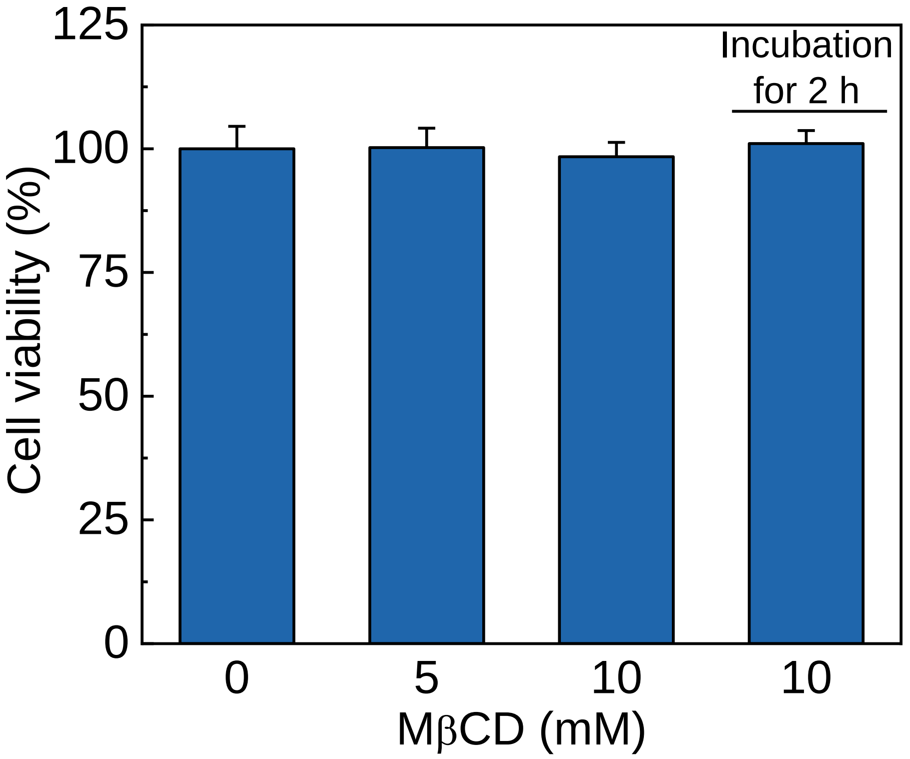

Fig. S11 Effect of acute cholesterol depletion on cell viability. CHO-K1 cells were tested for viability using MTT assay upon treatment with increasing concentrations of MβCD. To explore the kinetics of the reorganization of actin cytoskeleton upon acute cholesterol depletion, cells treated with 10 mM MβCD were washed with PBS and incubated in serum-free culture medium for 2 h (the last bar). Values are expressed as percentages of viability normalized to untreated cells. Data represent means ± SE of at least three independent experiments. See Materials and Methods for more details.

Supplemental Figure S12

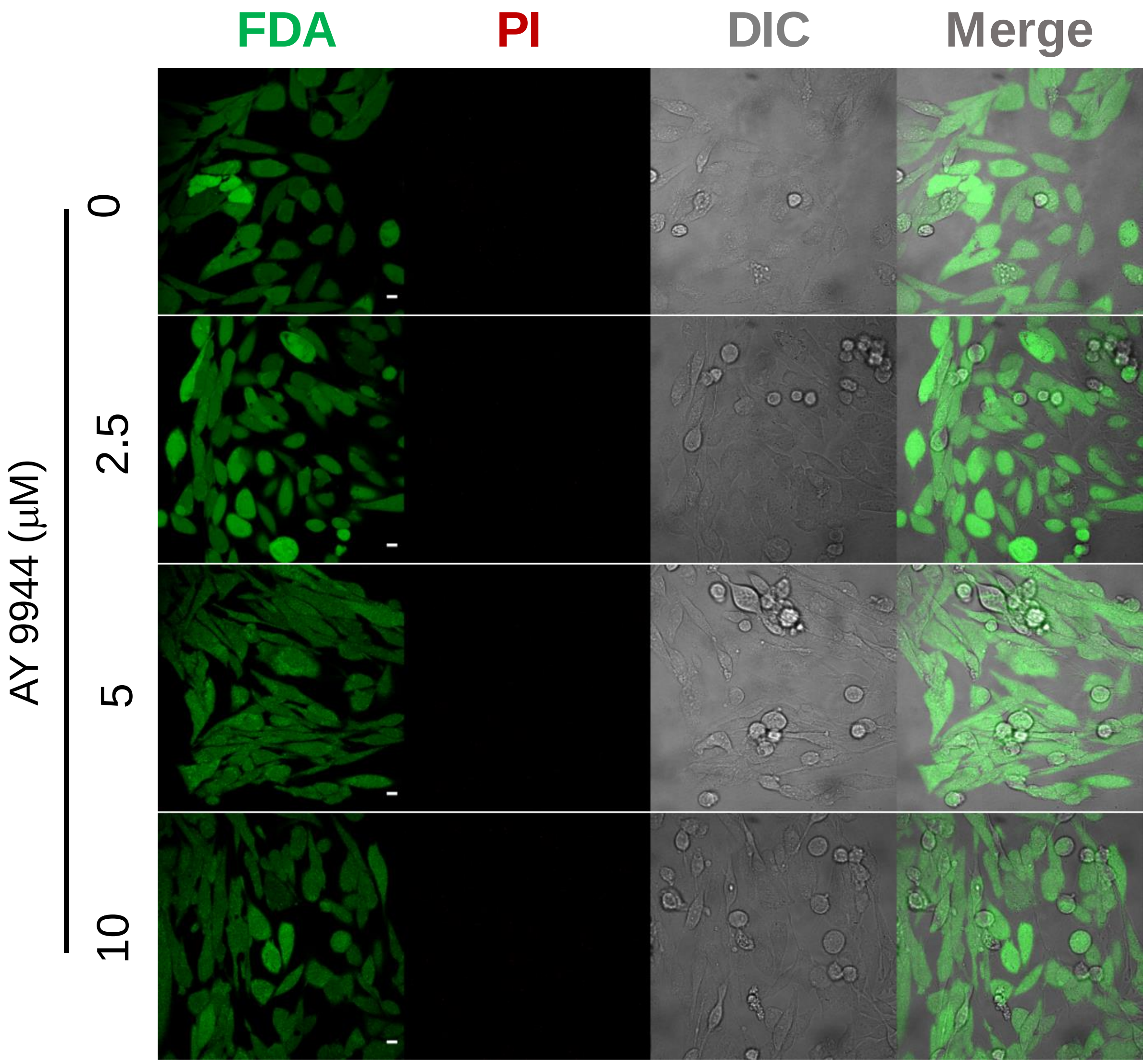

Fig. S12 Viability of CHO-K1 cells upon treatment with increasing concentrations of AY 9944 assessed by live-dead staining. The panels from left to right show FDA (green), PI (red) and DIC images of the same field. Merged images are shown in the panel on the extreme right. The scale bars represent 10 µm. See Materials and Methods for more details.
